# Timeline of Translational Formulation Technologies for Cancer Therapy: Successes, Failures, and Lessons Learned Therefrom

**DOI:** 10.3390/pharmaceutics12111028

**Published:** 2020-10-28

**Authors:** Alexandre Pérez-López, Cristina Martín-Sabroso, Ana Isabel Torres-Suárez, Juan Aparicio-Blanco

**Affiliations:** 1 Department of Pharmaceutics and Food Technology, Faculty of Pharmacy, Complutense University of Madrid, 28040 Madrid, Spain; alexap03@ucm.es (A.P.-L.); crmartin@ucm.es (C.M.-S.); juan.aparicio.blanco@ucm.es (J.A.-B.); 2 Institute of Industrial Pharmacy, Complutense University of Madrid, 28040 Madrid, Spain

**Keywords:** translational medicine, drug delivery, market approval, nanomedicine, microspheres, implants, medical devices, peptide-based therapy, chemotherapy, radiotherapy

## Abstract

Over the past few decades, the field of cancer therapy has seen a significant change in the way in which formulations are designed and developed, resulting in more efficient products that allow us to ultimately achieve improved drug bioavailability, efficacy, and safety. However, although many formulations have entered the market, many others have fallen by the wayside leaving the scientific community with several lessons to learn. The successes (and failures) achieved with formulations that have been approved in Europe and/or by the FDA for the three major types of cancer therapy (peptide-based therapy, chemotherapy, and radiotherapy) are reviewed herein, covering the period from the approval of the first prolonged-release system for hormonal therapy to the appearance of the first biodegradable microspheres intended for chemoembolization in 2020. In addition, those products that have entered phase III clinical trials that have been active over the last five years are summarized in order to outline future research trends and possibilities that lie ahead to develop clinically translatable formulations for cancer treatment.

## 1. Introduction

Cancer is one of the most common causes of morbidity and mortality worldwide. Of all the types of cancer, lung cancer is the most commonly diagnosed cancer, making up 11.6% of all cases, and the leading cause of cancer-related death (18.4% of all cancer deaths), closely followed by female breast cancer and colorectal cancers in terms of incidence and mortality, respectively [1].

Cancer therapy has been able to overcome different challenges over time. A lot of resources have been invested in developing new therapeutic agents and setting up secure and effective treatments [2,3]. Of the available therapeutic options for cancer treatment, the most common options used nowadays are surgery, peptide-based therapy, chemotherapy, and radiotherapy [4]. These can be used as a first-line treatment in advanced cancers or in combination as either a neoadjuvant or an adjuvant treatment. As a neoadjuvant therapy, peptides, chemotherapeutics, and radiotherapeutics are administered prior to surgery to reduce the size of the tumor. As an adjuvant therapy, the administration occurs after surgery to lower the risk of cancer recurrence.

The mechanism of action differs depending on the type of therapy. Peptides aim to interfere with biological processes to stop or slow down the growth of cancer and possess many advantages, such as high specificity, low toxicity, and good biocompatibility [5,6]. The best classical example of the application of peptides in cancer treatment is the use of gonadotropin-releasing hormone (GnRH) agonists. The prolonged activation of GnRH receptors by GnRH agonists leads to desensitization through receptor downregulation to reduce the secretion of gonadotropin hormones, which results in inhibition of the growth of certain hormone-dependent tumors [7]. The main drawbacks of peptides are the extremely low half-life and the low bioavailability once administered. Therefore, in order to maintain an effective therapeutic plasma concentration, the frequency of doses needs to be increased, which inevitably leads to poor patient compliance, potential side effects, and an increase in the cost of therapy [8,9].

Chemotherapeutics focus mainly on the interruption of cell division in cancer cells using cytotoxic agents. Depending on the type of mechanism of action and chemical structure, chemotherapeutics are classified into different groups, namely alkylating agents (e.g., carmustine and cisplatin), antimetabolites (e.g., cytarabine), antitumor antibiotics (e.g., daunorubicin and doxorubicin), topoisomerase inhibitors (e.g., irinotecan), and mitotic inhibitors (e.g., docetaxel, paclitaxel, and vincristine). The main inconvenience of this type of treatment is the nonspecific distribution and inability to exclusively target cancer cells, which account for their side effects, such as gastrointestinal tract lesions, a compromised immune system, hair loss, nausea, and cardiotoxicity. These side effects lower their therapeutic index and ultimately limit the dose that can be administered to patients, often resulting in treatment failure and cancer relapse [10].

Finally, there are two common procedures regarding radiotherapy. The first is external beam radiotherapy, in which damage to cancer cells is induced by the application of ionizing radiation using an external device. The second is brachytherapy, in which a radioactive source is inserted into the body to deliver the dose to a small, well-defined anatomy [11]. Similar to chemotherapy, the lack of tumor selectivity is generally associated with short-term toxicity and long-term consequences that are very similar to chemotherapy’s side effects. For instance, acute toxicity, such as mucositis, generally heals within weeks to months while late-onset effects such as fibrosis are generally considered to be irreversible and progress over time [12].

In this context, technological advances in formulations can help to prolong overall survival, increase the response rate, and reduce the systemic toxicity of conventional anticancer therapies by improving some therapeutics’ inherent properties such as poor aqueous solubility and in vivo instability, as well as increasing the selectivity of anticancer therapeutics. Aware that new formulations for cancer therapy with improved bioavailability, efficacy, and safety constitute the holy grail, we describe herein the successes and failures of, and lessons learned from, marketed formulations that have been approved in Europe and/or by the FDA as drug products or medical devices. In addition, those nonmarketed products that have entered phase III clinical trials are summarized in order to outline future research trends and possibilities that lie ahead to develop clinically translatable formulations for cancer treatment. The main body of the review is divided into four distinct time periods, each of which deals with peptide-based therapy, chemotherapy, and radiotherapy separately.

## 2. 1990s: The First Steps

During this period, two different formulation strategies aimed at increasing the therapeutic index of cancer therapies received market approval. On the one hand, extended-release formulations were designed to accurately control release rates over prolonged periods of time, achieving optimal drug concentration–time profiles and, therefore, a lower frequency of administration and less undesirable effects caused by the fluctuation in drug levels in the blood [13,14]. During this decade, various biomolecules were successfully encapsulated within subcutaneous or intramuscular long-acting formulations such as microparticles or implants (Table 1).

**Figure 1:**
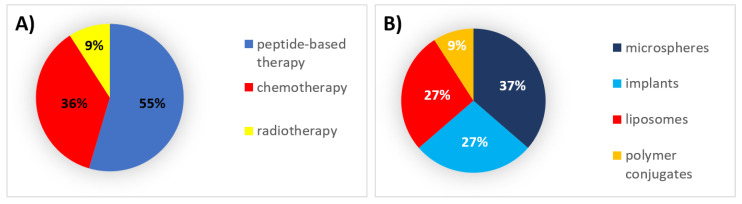
(**A**) Analysis of drug products and medical devices approved in the 1990s classified by the type of therapy. (**B**) Analysis of drug products and medical devices approved in the 1990s classified by the type of formulation.

On the other hand, researchers started to explore the potential of modifying the biodistribution of cancer therapeutics in order to increase the degree of accumulation at the target site and reduce adverse effects in patients [15,16,17]. This objective can be achieved by administering drugs directly to the tumor site using biodegradable polymer wafers, particularly when access to the target site is hindered when administered systemically, as in the case of brain tumors due to the blood–brain barrier [18,19]. Another method for controlling the biodistribution of anticancer agents is the intravascular administration of carriers that exploit the inherent characteristics of the tumor environment to increase the degree of accumulation of drugs within the target site, which is known as passive targeting. As a result of the enhanced permeability and retention (EPR) effect, therapeutic agents accumulate preferentially in neoplastic tissues when their circulation time in the bloodstream is prolonged [20]. The EPR effect is a phenomenon that describes the extravasation and accumulation of macromolecules or nanoparticles in neoplastic tissues [21]. This effect stems from both the peculiarities of the tumor’s neovasculature, which is composed of poorly aligned and defective endothelial cells with wide fenestrations that become leaky and permeable enough to allow therapeutics to reach the tumor’s surroundings, and the aberrant lymphatic architecture, wherein the high tissue pressure causes the drainage to be impaired, which ultimately helps to retain permeated nanoparticles and macromolecules [22,23]. The size of colloid carriers dictates their in vivo fate following intravascular administration. In this respect, while colloids above 5 μm in size can cause the occlusion of blood vessels, the size of carriers in the nanometer range determines their plasma circulation time, since particle size positively correlates with the extent of recognition by the reticuloendothelial system (RES). As a result, research efforts started to focus on accurately controlling the size of colloid carriers depending on the therapeutic aim. Microspheres pursuing embolization (the action of deliberately obstructing the blood flow in a particular vessel) have been approved for the treatment of hepatocellular carcinoma. Nanomedicines that take advantage of the EPR effect have been approved for other types of cancer,

During this decade, 55% of all formulations that received authorization were peptides, which were formulated mainly within microspheres and implants. Thirty-six percent (36%) were chemotherapeutic agents formulated mainly in liposomes (Figure 1).

### 2.1. Authorized Formulations for Peptide-Based Therapy

Due to the pharmacokinetics of peptide therapeutics for cancer treatment, products authorized during this period were formulated as either prolonged-release systems or polymer conjugates for intravascular administration [24].

Firstly, parenteral long-acting systems, after subcutaneous or intramuscular injections, form depots in order to slowly release the payload over an extended period and, consequently, reduce the need for frequent administration. Therefore, hormone analogs have benefited from these systems [24,25]. Leuprolide, goserelin, triptorelin, and buserelin are short synthetic peptide analogs of GnRH that inhibit estrogen and androgen synthesis and are used predominantly as an androgen deprivation therapy for advanced prostate cancer. Microspheres and implants, generally formulated with poly(lactic acid) (PLA) or poly(lactic-co-glycolic acid) (PLGA), which are biodegradable polymers with excellent biocompatibility, low immunogenicity, and good mechanical properties, have been successfully used to formulate these analogs [26,27]. The biodegradation rate of PLGA copolymers depends on the molar ratio of the lactic and glycolic acids in the polymer chain; the biodegradation rate increases as the lactic: glycolic molar ratio decreases [28]. There are three long-acting formulations that use microspheres and two formulations that use implants that have received authorization in either the United States or Europe during this decade and have provided the basis for further approvals in the following decades.

Decapeptyl^®^ and Lupron Depot^®^ are two intramuscular microsphere-based depot formulations that entrap triptorelin and leuprolide, respectively, and have been approved for the treatment of advanced prostate cancer. Decapeptyl^®^ was approved in 1986 in Europe and is also marketed under the trade names of Trelstar^®^ in the United States (2000) and Pamorelin^®^ in Europe [26]. With a mean particle size of ~40 μm, Decapeptyl^®^ is currently available in one-month (3.75 mg), three-month (11.25 mg), and six-month (22.5 mg) formulations [29,30]. There is no information in the datasheet about the molar ratio of the PLGAs; however, Skidmore et al. recently characterized the PLGA in the Trelstar^®^ formulations and obtained lactic/glycolic molar ratios of 57:43, 78:22, and 80:20 for the one-, three-, and six-month formulations, respectively [31]. Lupron Depot^®^ was approved in 1989 by the FDA. It has a mean particle size of 8 μm and is available in one-month (7.5 mg), three-month (22.5 mg), four-month (30 mg), and six-month (45 mg) formulations. The one-month formulation is composed of PLGA (75:25 lactic/glycolic molar ratio) and the three-, four-, and six-month depot injections are composed of PLA [32]. In one clinical trial extracted from the FDA label for Lupron Depot^®^, the six-month formulation suppressed serum testosterone to castrate levels (<50 ng/dL) from Week 4 to Week 48.

The third microsphere-based formulation is Sandostatin LAR^®^, an intramuscular long-acting dosage form that encapsulates octreotide acetate within PLGA (55:45 lactic/glycolic molar ratio) microspheres of about 50 μm in size. It was approved in 1998 in Europe for the treatment of recurrent, inoperable, or metastatic gastrointestinal neuroendocrine tumors [33]. Sandostatin LAR^®^ is available as a monthly formulation in three different doses. It is also approved by the FDA but only for other noncancer indications.

Apart from microspheres, a subcutaneous implant of goserelin, Zoladex^®^, was approved in 1989 by the FDA (and shortly thereafter in Europe) for hormonal manipulation of advanced carcinoma of the prostate (in combination with flutamide in the United States) and of breast cancer. Zoladex^®^ consists of implants of PLGA in the form of a rod of about 1 mm in diameter and 3–6 mm in length for the one- or three-month delivery of 3.6 and 10.8 mg of goserelin, respectively. The one-month formulation is composed of PLGA with a 50:50 lactic/glycolic molar ratio, whereas the three-month formulation is composed of PLGA with a 95:5 lactic/glycolic molar ratio. Seven years later, another PLGA (75:25 lactic/glycolic molar ratio) subcutaneous implant containing buserelin was authorized in Europe for the treatment of hormone-dependent prostate cancer and marketed under the trade names of Suprefact Depot^®^ and Profact Depot^®^. It is currently available in two-month (6.6 mg) and three-month (9.9 mg) formulations [26]. This buserelin implant has not been approved by the FDA.

Secondly, polymer conjugates have been developed for the purpose of improving the short elimination half-life and reducing the high immunogenicity of distinct biologics such as enzymes. Oncaspar^®^ (pegaspargase) is a polymer conjugate that was authorized in 1994 by the FDA (and in 2016 by the EMA) as part of a multiagent regimen for the treatment of acute lymphoblastic leukemia (ALL), in which it is a first-line treatment following intravenous or intramuscular administration every two weeks [34]. It consists of 69–82 molecules of 5 kDa monomethoxy-polyethylene glycol (mPEG) linked to the enzyme l-asparaginase through a succinimidyl succinate linker. Pegylation improves the pharmacokinetic profile and immunogenicity [35] of l-asparaginase: Oncaspar^®^ shows a longer elimination half-life than the native enzyme without affecting the enzymatic properties [34].

### 2.2. Authorized Formulations for Chemotherapy

During this decade, new technologies based on a modification of the biodistribution of chemotherapeutics were authorized under the assumption that this could reduce their nonspecific toxicity. One such strategy consists of locally administering chemotherapeutics-loaded polymer implants. However, factors such as the localization, grade, and stage of the tumor are essential to the success of local treatment. Implants containing chemotherapeutics are uncommon due to the requirement of a high dose of these cytotoxic drugs, which puts the patient at risk in the case of system failure with abrupt liberation of the content [36]. Gliadel^®^, which received authorization from the FDA in 1996 and from the EMA in 1999, is an implant of about 1.45 cm in diameter and 1 mm thick used in the treatment of glioblastoma multiforme upon intracranial implantation during resection surgery. It contains carmustine and the biodegradable copolymer polifeprosan 20, which consists of poly[bis (*p*-carboxyphenoxy)] propane and sebacic acid (in a 20:80 molar ratio), that extends the release of carmustine over three weeks. Gliadel^®^ minimizes the potential risk deriving from the formulation’s breakdown by dose fractionation into eight 7.7 mg wafers [37,38]. Gliadel^®^ prolonged the median survival of patients with a newly diagnosed high-grade glioma by 2.2 months [39].

Alternatively, chemotherapeutics have been loaded into nanocarrier-based systems for intravenous administration. Once in contact with the biological milieu, the surface of the carrier is rapidly covered by a corona of adsorbed serum proteins, a process known as opsonization. Afterwards, the RES recognizes the protein corona and, as a result, drug carriers are internalized and cleared from systemic circulation [40,41,42]. Covalent linkage to polyethylene glycol (PEG), which is known as stealth technology, makes drug carriers less susceptible to recognition by the RES, probably due to its high hydrophilicity, electrical neutrality, and chain flexibility. Moreover, it also changes the hydrodynamic size reducing their renal clearance and, therefore, prolonging their circulation time [43]. One liposomal formulation exploiting stealth technology was approved during this period by both the FDA (1995) and the EMA (1996) under the trade names Doxil^®^ and Caelyx^®^, respectively. It was approved for the treatment of AIDS-related Kaposi’s sarcoma (1995) and, later, for recurrent ovarian cancer (1998) and multiple myeloma (2003) [44]. Moreover, in Europe, it was authorized for the treatment of metastatic breast cancer. It is a long-circulating pegylated liposomal formulation with an approximate mean size in the range of 80–90 nm and is composed of hydrogenated soy phosphatidylcholine, cholesterol, and 1,2-distearoyl-sn-glycero-3-phosphoethanolamine-*N*-(methoxy-PEG) in a 56:39:5 molar ratio [44]. It shows a longer plasma half-life, a 60-fold increased AUC, and lower clearance compared with free doxorubicin [45]. Liposomal doxorubicin also reduces the risk of suffering from cardiotoxicity, a broadly acknowledged dose-limiting side effect of free doxorubicin [46,47]. In one clinical trial extracted from the FDA label, the efficacy of Doxil^®^ was evaluated in combination with bortezomib in multiple myeloma. The primary outcome measure was time to progression (TTP), defined as the time from randomization to the first occurrence of progressive disease or death due to progressive disease. Doxil^®^ and bortezomib demonstrated significant improvement in TTP (282 days) compared to bortezomib alone (197 days).

Apart from that, two nonstealth liposomes received marketing authorization during this period. In 1996, DaunoXome^®^, a 45-nm-sized, nonpegylated, and daunorubicin-loaded unilamellar liposomal formulation, composed of distearoyl phosphatidylcholine and cholesterol (in a 2:1 molar ratio), was approved by the FDA and in Europe for the treatment of AIDS-related Kaposi’s sarcoma. Despite the absence of PEG, clinical pharmacokinetic studies have demonstrated that the AUC following DaunoXome^®^ administration was 36-fold higher than that following conventional daunorubicin administration (375.3 vs. 10.33 μg·h/mL) [48,49,50]. The third type of carrier-based technology was released in the 1990s in order to take advantage of passive targeting: DepoFoam^TM^, a multivesicular liposomal formulation. Morphologically, it has been described as a spheroid with aqueous internal cameras limited by a single bilayer lipid membrane [43,51]. DepoCyt^®^, authorized in 1999 by the FDA for the treatment of lymphomatous meningitis following intrathecal administration, is a sustained-release formulation of cytarabine that exploits DepoFoam^TM^ technology. These liposomes are composed of cholesterol, triolein, dioleoylphosphatidylcholine, and dipalmitoylphosphatidylglycerol and are bigger than standard unilamellar or multilamellar liposomes (3–30 μm). A version of this product, called DepoCyte^®^, was authorized by the EMA in 2001; however, in December 2016 at the manufacturing site, several batches failed standard quality tests and the product was withdrawn from use.

### 2.3. Authorized Formulations for Radiotherapy

Selective internal radiation therapy (SIRT) radioembolization consists of the intra-arterial injection of micron-sized embolic particles loaded with a radioisotope in order to deliver high focal doses of radiation to cancers, especially hepatocellular carcinoma (HCC), increasing the dose of radiation in the tumor with minor radiation-induced hepatic damage [52,53,54]. Yttrium-90 (^90^Y) is the most common radioisotope used for radioembolization. This treatment is based on the fact that tumoral liver lesions are generally vascularized via the hepatic artery, whereas the healthy liver is almost completely excluded from arterial vascularization and blood is supplied via the portal vein. Therasphere^®^ was approved in 1999 by the FDA as a medical device and received a Conformité Européenne (CE) mark in 2014 for the radioembolization of HCC. The Conformité Européenne (CE) marking is a legal prerequisite of placing a medical device on the market in the European Union and indicates that a medical device conforms with the applicable safety and performance requirements set out in the relevant European Medical Devices Directives and Regulations. Therasphere^®^ consists of 20–30-μm-sized nonbiodegradable glass microspheres containing yttrium-90. It is available in six doses ranging from 3 to 20 GBq. Various clinical studies have shown that Therasphere^®^ is a safe and effective modality for treating patients that can limit the progression of HCC [55,56].

## 3. 2000s: The Field Matures

From the beginning of the 21st century, the field matured through the diversification of new technologies in the direction of the strategies described in the previous section. Concerning the development of extended-release platforms, two distinct nonbiodegradable solid implants able to prolong peptide release over a year (including the first osmotic-driven technology) were marketed during this decade (Table 2). Moreover, the Atrigel^®^ technology entered the arena as an alternative to solid implants, which forms implants in situ upon phase separation by solvent exchange [51]. With regard to delivery systems for the modification of the biodistribution of cancer therapeutics, new intravascular carrier-based technologies, such as nanoparticle albumin-bound (nab) technology, were introduced to improve the efficiency of nanoparticles and further reduce the side effects of chemotherapy (Table 2) [57,58]. Furthermore, the first microspheres for chemoembolization were CE-marked during this decade. Chemoembolization refers to the technique of injecting chemotherapeutic agents into the feeding arteries of a tumor along with particles designed to cause embolization. Transarterial chemoembolization (TACE) is a first-line treatment for the intermediate stage of HCC [59]. However, the possibility of the diffusion of and systemic toxicity due to the chemotherapeutics used in solution in conventional TACE [60,61] required the development of drug-eluting microspheres (DEM) as better embolization agents in TACE (DEM-TACE), as they allow for chemotherapeutics to be retained selectively in HCC.

**Figure 2:**
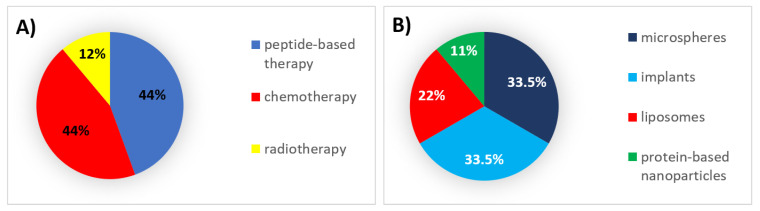
(**A**) Analysis of drug products and medical devices approved during the 2000s classified by the type of therapy. (**B**) Analysis of drug products and medical devices approved during the 2000s classified by the type of formulation.

During this decade, 44% of all formulations that received authorization were peptides and chemotherapeutic agents, which were mainly encapsulated in implants and microspheres, respectively (Figure 2).

### 3.1. Authorized Formulations for Peptide-Based Therapy

The success of prolonged-release therapeutics encapsulating synthetic hormones in the previous decade led to the approval of delivery systems able to extend peptide release over longer periods to treat prostate cancer. Two subcutaneous solid implants, Viadur^®^ (2000) and Vantas^®^ (2004), were received authorization by the FDA. Both are nonbiodegradable reservoir drug delivery systems designed to deliver leuprolide and histrelin, respectively, for 12 months. Viadur^®^ is formulated with the osmotic-driven Duros^®^ implant technology to deliver leuprolide with zero-order release kinetics over one year. The implant consists of a cylindrical titanium alloy reservoir capped on one end by a rate-controlling membrane through which water from the body is imbibed in response to the osmotic gradient created by an osmotic material (namely, sodium chloride) that expands, which ultimately forces leuprolide to be released through an orifice placed at the other end of the implant. The external dimensions of the implant are a diameter of 4 mm and a length of 45 mm [62]. Vantas^®^ is a cylindrical implant (measuring 3.5 cm in length and 3 mm in diameter) containing a histrelin inner core surrounded by a flexible, polymethacrylate-based hydrogel reservoir that provides the diffusion-controlled release of histrelin over one year upon subcutaneous implantation [63,64]. Neither of these implants has ever received marketing authorization in Europe.

As an alternative to solid implants, Eligard^®^ is an in-situ-forming implant that exploits the Atrigel^®^ technology to formulate leuprolide and was approved in 2002 by the FDA (and shortly thereafter in Europe) for the treatment of prostate cancer following subcutaneous administration. It is an extended-release biodegradable formulation wherein leuprolide is mixed in a polymeric solution of PLGA dissolved in the water-miscible solvent *N*-methyl-2-pyrrolidone to form an injectable dispersion. The drug–polymer combination is then administered subcutaneously, where it forms a PLGA depot upon solvent diffusion to aqueous body fluids that slowly releases leuprolide at a controlled rate as the polymer is degraded. The rate of release of leuprolide can be controlled by varying the molecular weight of the polymer and the amount of solvent. Eligard^®^ is commercially available in one-month (7.5 mg), three-month (22.5 mg), four-month (30 mg), and six-month (45 mg) formulations. The monthly formulation is composed of PLGA in a 50:50 lactic/glycolic molar ratio, while the three-, four-, and six-month formulations are composed of PLGA in 75:25, 75:25, and 85:15 lactic/glycolic molar ratios, respectively. Data suggested that all formulations have comparable efficacy and safety; however, the Eligard^®^ six-month formulation was found to be more cost-effective [65,66]. The AGL0205 study using Eligard^®^ 45 mg evaluated the achievement and maintenance of castrate serum testosterone suppression over the duration of therapy. The treatment was found to achieve a reduction in testosterone from 367.7 ng/dL to 12.6 ng/dL.

Finally, in 2009, the EMA authorized Mepact^®^ for the treatment of high-grade, nonmetastatic osteosarcoma after macroscopically complete surgical resection in children, adolescents, and young adults following intravenous infusion. It is used in combination with postoperative multiagent chemotherapy. Mepact^®^ is a 100-nm-sized non-PEG multilamellar liposomal formulation containing mifamurtide, a synthetic analog of the muramyl tripeptide with immunomodulatory properties. It represents the first time that a peptide analog was encapsulated within liposomes for cancer therapy. Dioleoyl-phosphatidylserine and 1-palmitoyl-2-oleoyl-phosphatidylcholine (in a 3:7 molar ratio) were used in the preparation of these liposomes. In 2009, Chou et al. published a paper describing a phase III trial (*n* = 91) of liposomal mifamurtide in patients with osteosarcoma. The results showed a 16% increase in the survival rate for patients who received liposomal mifamurtide (*n* = 46) compared with those who did not (*n* = 45) [67]. This product has not been approved by the FDA.

### 3.2. Authorized Formulations for Chemotherapy

During this decade, two additional chemotherapeutic-loaded, nanocarrier-based systems for intravenous administration were marketed. Nonpegylated doxorubicin-loaded liposomes (Myocet^®^) received marketing authorization by the EMA in 2000 under the assumption that the nonpegylation could reduce the palmar–plantar erythrodysesthesia (commonly known as hand–foot syndrome) observed upon long-term exposure to Doxil^®^. This disorder causes swelling, redness, blisters, inflammation, and pain erythema on the palms of the hands and the soles of the feet, hence deteriorating patients’ quality of life [68,69,70,71]. This formulation was approved as a first-line treatment for metastatic breast cancer in combination with cyclophosphamide. These liposomes are about 150 to 250 nm in size and contain cholesterol and egg phosphatidylcholine (in a 45:55 molar ratio). This product has never received FDA approval.

The first chemotherapeutic to use nab technology received approval by the FDA in 2005 and by the EMA in 2008. Abraxane^®^ is a 130 nm, paclitaxel-loaded, albumin-based nanocarrier. Abraxane^®^ is used for the management of metastatic breast cancer, in combination with carboplatin as a first-line treatment of non-small-cell lung cancer (2012), and in combination with gemcitabine as a first-line treatment for metastatic adenocarcinoma of the pancreas (2013). Traditionally, paclitaxel formulations required certain solvents, such as Cremophor EL^®^, to increase their solubility in water, which has been related to hypersensitivity, nephrotoxicity, and neurotoxicity, and consequently requiring a reduction in dose [72]. Abraxane^®^ solves this toxicity problem by avoiding the use of Cremophor EL^®^. In clinical trials, it showed a higher volume of distribution, no differences in terminal half-lives, lower toxicity, and higher intratumor concentrations compared with traditional paclitaxel formulations, hence demonstrating that nab technology allows Abraxane^®^ to increase the therapeutic index of paclitaxel [73,74,75]. In one clinical trial extracted from the FDA label, patients in the Abraxane^®^ treatment arm had a statistically significantly higher reconciled target lesion response rate (21.5%) compared with patients in the paclitaxel injection treatment arm (11.1%).

Regarding intra-arterial proceedings, carrier-based systems that combine drug delivery with embolization were developed to limit the nonspecific biodistribution of chemotherapeutics. DEMs are nonresorbable embolic beads loaded with cytotoxic agents that, despite having been granted a CE marking in Europe, have not been validated by the FDA for drug loading. They lodge preferentially in the tumor microvasculature, wherein they cause ischemia and necrosis while releasing the embedded chemotherapeutic drug in a sustained manner, which enables local chemotherapy to be delivered and reduces the drug’s systemic toxicity [61,76,77]. During this decade, two types of DEMs were approved as medical devices to treat HCC. DC Bead^®^ was the first one to be approved (2003) and is fabricated by free radical polymerization of poly(vinyl alcohol) with modification of *N*-acryloyl-amino acetaldehyde, 2-acrylamido-2-methylpropane sulfonate sodium salt, and cellulose acetate butyrate. DC Bead^®^ was approved for loading with doxorubicin and irinotecan. It is available in various sizes (70–150, 100–300, 300–500, and 500–700 µm). In clinical studies, the use of 100–300 μm doxorubicin-loaded DC Bead^®^ decreased the rate of occurrence of adverse events and complications in comparison with larger microspheres as they allowed for a more distal embolization and deeper penetration into the tumor [78]. Various studies with a long-term follow-up have shown that TACE using DC Bead^®^ loaded with doxorubicin resulted in a large antitumoral effect and increased five-year survival rates [79]. The pharmacokinetic and safety profiles also suggest that high doses of doxorubicin can be used without systemic toxicity [80,81,82].

The second type of DEM, HepaSphere^®^, was released by 2005, is fabricated using a sodium acrylate alcohol copolymer, and is available in a dry diameter of 30–60, 50–100, 100–150, and 150–200 µm, corresponding to a hydrated diameter of 120–240, 200–400, 400–600, and 600–800 µm, respectively. In one study using HepaSphere^®^ microspheres, Malagari et al. confirmed the safety and effectiveness of the drug-eluting technology with a low degree of systemic exposure to doxorubicin [83].

### 3.3. Authorized Formulations for Radiotherapy

SIRT using microspheres was approved during the previous decade as a technique for circumscribing irradiation to neoplastic tissue while sparing healthy liver tissue to the greatest extent possible. In 2002, another intra-arterial formulation of microspheres loaded with ^90^Y, under the tradename SIR-Spheres^®^, was authorized by the FDA and CE-marked as a medical device for the treatment of unresectable metastatic liver tumors from primary colorectal cancer with adjuvant chemotherapy with floxuridine. The recommended dose ranges between 2.0 and 3.0 GBq. In comparison with TeraSphere^®^, SIR-Spheres^®^ are composed of an ion-exchange resin instead of glass [84], slightly larger (20–40 μm), and less dense, and, thus, associated with a greater occlusion of the blood vessels in the liver [56].

## 4. 2010s: The End of the Beginning

During this decade, the application of previously authorized technologies was consolidated into distinct cancer therapeutics (Table 3). Concerning the development of extended-release platforms during this decade, new approvals were limited to a single solid implant and to a polymer conjugate.

With regard to delivery systems for the modification of the biodistribution of cancer therapeutics, three additional liposomal formulations received marketing authorization, including the second stealth liposome (after Doxil^®^’s approval in the 1990s) and the very first liposome coencapsulating two distinct chemotherapy agents, which enables the simultaneous delivery of both drugs [50]. Moreover, in 2018, another milestone was achieved with the approval of the first micellar formulation for cancer therapy. Additional breakthroughs stem from the pioneering approval of inorganic-based nanoparticles. In fact, during this decade, two different products using inorganic nanoparticles were authorized as medical devices for cancer therapy: Hensify^®^ as a radio enhancer in radiotherapy and NanoTherm^®^ for magnetic hyperthermia [85].

**Figure 3:**
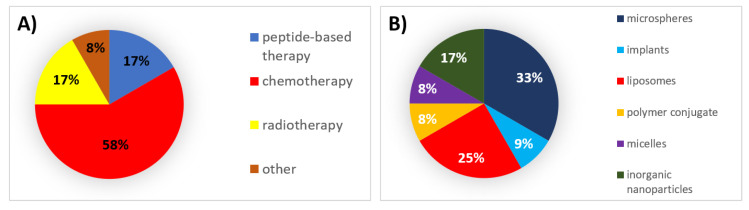
(**A**) Analysis of drug products and medical devices approved during the 2010s classified by the type of therapy. (**B**) Analysis of drug products and medical devices approved during the 2010s classified by the type of formulation.

During this decade, 58% of all formulations that received authorization were chemotherapeutic agents, for which microspheres and liposomes were the mainly used types of formulations (Figure 3).

### 4.1. Authorized Formulations for Peptide-Based Therapy

Twenty-four years after Oncaspar^®^ was first approved, a new polymer conjugate of l-asparaginase received marketing authorization from the FDA: Asparlas^®^. It is a formulation containing calaspargase pegol (69–82 molecules of 5 kDa mPEG linked to l-asparaginase) for the treatment of ALL following intravenous administration [86]. In comparison with Oncaspar^®^, Asparlas^®^ was developed using a more stable succinimidyl carbamate linker between the PEG and l-asparaginase that results in a longer period of serum asparaginase activity over pegaspargase with similar toxicities, which enable the dosing frequency to be extended to once every three weeks [87,88]. This polymer conjugate has yet to be approved in Europe. In one clinical trial extracted from the FDA label, the determination of efficacy was based on a demonstration of the achievement and maintenance of serum asparaginase activity above the level of 0.1 U/mL by intravenous administration of Asparlas^®^ every three weeks. The results showed that 123 of the 124 patients maintained asparaginase serum levels >0.1 U/mL at Weeks 6, 12, 18, 24, and 30.

Leptoprol^®^, a subcutaneous long-acting solid implant of leuprolide, was authorized in 2015 in Europe for the treatment of advanced prostate cancer. Leptoprol^®^ is a 10-mm-long cylinder-shaped implant. There is only one formulation available (5.25 mg of leuprolide acetate). It contains PLA, which extends the release of leuprolide over three months. This implant has yet to be approved by the FDA.

### 4.2. Authorized Formulations for Chemotherapy

Three new liposomal formulations were approved during this decade for intravenous administration. The first one is Marqibo^®^, which is a vincristine nonpegylated liposomal formulation that was approved in 2012 by the FDA for the treatment of adult patients with Philadelphia-chromosome-negative ALL. Marqibo^®^ has an approximate size of 100 nm and is composed of sphingomyelin and cholesterol (in a 60:40 molar ratio), which contribute to low protein binding, resulting in a prolonged circulation time for the liposome [43]. Traditionally, the use of free vincristine has been limited by rapid initial plasma clearance and neurotoxicity, which make vincristine a good candidate to be encapsulated in liposomes [89,90]. Clinical trials demonstrate Marqibo^®^’s safety, tolerability, and promising activity as well as slower clearance (345 mL/h) and higher AUC in comparison with vincristine administered in solution (11.34 mL/h) [91]. Marqibo^®^ has not been approved in Europe. The second liposomal formulation is Onivyde^®^, which was approved in 2015 by the FDA and in 2016 by the EMA as a second-line treatment for metastatic adenocarcinoma of the pancreas in combination with 5-fluorouracil and leucovorin. Onivyde^®^ is formulated with a water-soluble, semisynthetic irinotecan in a liposomal dispersion. The dispersion consists of 110-nm-sized unilamellar liposomes composed of dipalmitoyl phosphatidylcholine, cholesterol, and methoxy-terminated polyethylene glycol-distearoylphosphatidyl ethanolamine (in a 3:2:0.015 molar ratio) encapsulating irinotecan. Onivyde^®^ has been shown to provide highly efficient protection from rapid clearance and premature metabolism, a longer circulation time, and a 227-fold increase in AUC compared with nonliposomal irinotecan [92,93]. One clinical trial extracted from the FDA label compared the overall survival in patients treated with Onivyde^®^/5-fluorouracil/leucovorin with that in patients treated with 5-fluorouracil/leucovorin alone. The study demonstrated a statistically significant improvement in overall survival for the Onivyde^®^ group by 1.9 months and a 6% reduction in the number of deaths.

The third liposomal formulation is Vyxeos^®^, which was approved in 2017 by the FDA and in 2018 by the EMA in order to improve the existing 7 + 3 combinatory regime used to treat acute myeloid leukemia (AML), which consists of a continuous seven-day infusion of cytarabine plus an anthracycline (most commonly daunorubicin) on days 1–3 [94,95]. Previous efforts to improve the efficacy of the 7 + 3 regime, such as administration of high doses of cytarabine and daunorubicin or the addition of other chemotherapeutic agents, largely failed to improve outcomes [96]. In this context, Vyxeos^®^ is the first in a new class of liposomes that enable the simultaneous delivery of two drugs, cytarabine and daunorubicin, in a synergistically fixed 5:1 ratio to increase treatment efficacy with a lower cumulative dose. Due to their differential pharmacokinetics and biodistribution, the spatiotemporally controlled delivery of this optimal ratio cannot be achieved by any other approach than their coencapsulation within a single carrier. Vyxeos^®^ are 107-nm-sized liposomes composed of distearoylphosphatidylcholine, distearoylphosphatidylglycerol, and cholesterol (in a 7:2:1 molar ratio) [97,98]. In a phase III trial, Vyxeos^®^ prolonged overall survival by 3.61 months compared with conventional 7 + 3 chemotherapy, corresponding to a 31% reduction in the risk of death [99]. The safety profile of Vyxeos^®^ was found to be similar to that of 7 + 3 chemotherapy [95,96,100]. Apart from the aforementioned liposomal formulations, the first micellar formulation for cancer therapy was authorized in 2018 by the EMA for the treatment of the first relapse of platinum-sensitive epithelial ovarian cancer, primary peritoneal cancer, and fallopian tube cancer, and is given as an intravenous infusion in combination with carboplatin. Apealea^®^ is formulated with the XR17 technology to encapsulate paclitaxel. XR17 is a type of technology that utilizes a mixture of two isoforms of *N*-retinoyl-l-cysteic acid methyl ester sodium salt to form 20–30-nm-sized micelles. A study with a crossover design compared total and unbound paclitaxel concentrations in plasma after a 1 h infusion of Apealea^®^ with those after a 1 h infusion of albumin-bound paclitaxel at the same dose and concluded that both formulations, paclitaxel micellar and nab-paclitaxel, are clinically equivalent [101]. Apealea has yet to receive marketing authorization from the FDA.

Furthermore, changes in the composition and structure of DEMs have led to the marketing authorization of three new TACE microspheres as medical devices. First, Embozene Tandem^®^ was granted a CE marking in 2012. Embozene Tandem^®^ are small polymethacrylate microspheres coated with Polyzene-F that serve as a drug-releasing embolization system for doxorubicin and irinotecan. It is available in three highly calibrated sizes (40, 75, and 100 μm). Embozene Tandem^®^ showed promising results with a very fast loading ability, a favorable in vivo pharmacokinetic profile with sustained release during the first 24 h, an encouraging level of safety, and encouraging responses [102,103,104]. Second, LifePearl^®^, which was authorized in 2015 as a medical device in Europe, consists of chemoembolization microspheres made of a copolymer of PEG and diacrylamide that can be loaded with a wide range of chemotherapeutic agents (e.g., doxorubicin, irinotecan, idarubicin, and epirubicin). It is available in a variety of diameters ranging from 100 to 400 µm. As a PEG hydrogel that is biocompatible, LifePearl^®^ guarantees good compressibility and elasticity and maximizes the time in suspension, thus improving catheter deliverability, making the microsphere more resilient to stress and attrition, and providing more controlled chemoembolization with a uniform and distal distribution, allowing for a precise and efficacious occlusion of capillaries [105,106]. LifePearl^®^ is mixed with a nonionic contrast agent for visualization under fluoroscopy. The final DEM is DC Bead LUMI™, which was CE-marked in 2018. It consists of precisely calibrated, radiopaque, biocompatible, and nonresorbable microspheres. These microspheres range between 50 and 150 µm in size, are produced with polyvinyl alcohol, and contain a covalently bound radiopaque moiety which can be visualized by an X-ray-based imaging modality, such as computed tomography (CT). DC Bead LUMI™ is the first commercially available radiopaque drug-eluting bead in Europe that can be loaded with doxorubicin or irinotecan for the local treatment of tumors in patients with hepatocellular carcinoma or metastatic colorectal cancer, respectively.

### 4.3. Authorized Formulations for Radiotherapy

Radio enhancers are agents used to boost the effectiveness of radiotherapy. However, new types of radio enhancers need to be tested as the lack of complete control over energy deposition still causes unwanted damage to healthy tissues [107]. QuiremSpheres^®^, which received a CE marking in 2015, constitute the first biodegradable microspheres for SIRT. This formulation consists of 15–60-μm-sized microspheres pioneeringly made of PLA and containing Holmium-166 as a radio enhancer to treat unresectable liver malignancies, providing an alternative to ^90^Y microspheres with superior characteristics for imaging. Visualization of the microspheres is possible by single-photon emission computed tomography/computed tomography (SPECT/CT) and magnetic resonance imaging (MRI) [108]. QuiremSpheres^®^ have yet to be FDA-approved. Traditionally, radio enhancers have mainly been used in the treatment of liver cancers. However, hafnium oxide (HfO_2_) nanoparticles are a new class of radiation-enhancing nanoparticles for the treatment of solid tumors. Hensify^®^, which was CE-marked in 2019 for the treatment of locally advanced soft-tissue sarcoma following intratumoral administration, is an aqueous suspension of 50 nm crystalline hafnium oxide nanoparticles with a negatively charged phosphate coating [109,110]. Specifically, the interaction between the ionizing radiation and the hafnium facilitates a higher energy deposit as compared with ionizing radiation without interaction with hafnium; this results in the generation of significantly more electrons and increases the number of radiation-mediated cell deaths from standard radiation oncology [111,112,113]. Hensify^®^ has yet to be FDA-approved.

### 4.4. Other Authorized Products

Therapeutic hyperthermia is a type of treatment in which cancer cells are exposed to high temperatures (up to 40 °C) to induce thermal ablation of the tumor tissue and sensitize cancer cells to the effects of radiation and certain anticancer drugs [114]. Magnetic nanoparticles (MNPs), particularly superparamagnetic iron oxide nanoparticles (SPIONs), have recently been incorporated into the therapeutic arsenal in oncology. MNPs can transform electromagnetic energy from an external high-frequency field into heat because of the relaxation of their rotating magnetic moments, therefore inducing local heat in the tumor target and avoiding nonspecific damage [115]. In 2010, NanoTherm^®^ received authorization to be used in Europe. It is an aqueous colloidal dispersion of iron oxide nanoparticles of about 15 nm in size coated with aminosilane and used in focal thermal ablation of glioblastoma [116]. The MNPs are intratumorally administered and then heated under an alternating magnetic field generator [114,117]. Nanotherm^®^ has yet to be approved in the United States.

## 5. The Decades to Come

**Figure 4:**
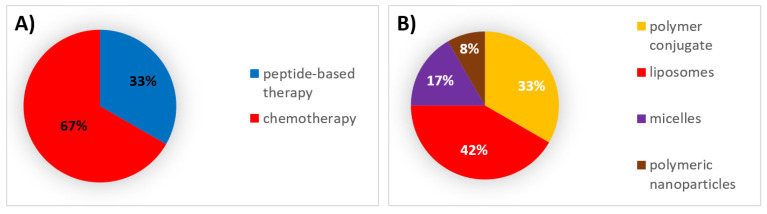
(**A**) Analysis of nonmarketed formulations for cancer therapy that have entered phase III clinical trials classified by the type of therapy. (**B**) Analysis of nonmarketed formulations for cancer therapy that have entered phase III clinical trials classified by the type of formulation.

Despite the limited number of formulations that have been able to be included in existing therapeutic regimes for multiple types of cancer, there are some innovative products under clinical trial based on marketed technologies for new therapeutics, new technologies that are being applied to well-known therapeutics, and even new delivery systems with new therapeutics. Table 4 summarizes the nonmarketed formulations that have entered phase III clinical trials and are eventually expected to receive approval in the decades to come. Of the drug products that have reached phase III clinical trials, the majority employ chemotherapeutic agents encapsulated within last-generation liposomes followed by peptide-based therapies administered as polymer conjugates (Figure 4).

Regarding peptide-based therapies, four different formulations have entered phase III clinical trials. Three are formulated as polymer conjugates, and one is formulated in liposomes to serve as a vaccine.

Two polymer conjugates, ADI-PEG 20 and PEGPH20, have been developed to extend the enzyme’s half-life. ADI-PEG 20 is a cloned form of arginine deiminase conjugated with PEG and has the potential to cause arginine deprivation in arginine-succinate synthetase 1-negative cancers. One phase III clinical trial has been completed (NCT01287585) using ADI-PEG 20 in HCC, showing that ADI-PEG 20 monotherapy did not confer an overall survival benefit in a second-line setting for HCC [118]. The phase III ATOMIC trial (NCT02709512) is currently recruiting patients with malignant pleural mesothelioma in order to evaluate the efficacy of the addition of ADI-PEG-20, given weekly following intramuscular administration, to the approved standard of care treatment (pemetrexed and cisplatin). PEGPH20 (pegvorhyaluronidase alfa) is a pegylated recombinant human hyaluronidase (rHuPH20) with the potential to degrade hyaluronan to ultimately remodel the tumor stroma [119]. In 2016, the HALO-109 phase III clinical trial (NCT02715804) was launched to evaluate the efficacy of PEGPH20, given once weekly following intravenous administration, in combination with Abraxane^®^ and gemcitabine in patients with hyaluronan-high previously untreated pancreatic ductal carcinoma. However, this clinical trial was prematurely terminated in 2020 by a sponsor decision due to a negative study outcome.

Polymer conjugates that extend the short half-life of peptide-based cancer immunotherapies have also entered phase III clinical trials. This is the case for pegilodecakin, a pegylated recombinant human interleukin 10 with the potential to activate tumor-infiltrating CD8^+^ T cells. [120]. The efficacy of pegilodecakin administered subcutaneously in combination with FOLFOX therapy (a combination chemotherapy regimen that includes folinic acid, fluorouracil, and oxaliplatin and is usually used to treat colorectal cancer) versus FOLFOX alone was compared in patients with metastatic pancreatic cancer who were previously on a gemcitabine regimen in the phase III Sequoia trial (NCT02923921). The Sequoia study was completed in 2020 and results are pending.

Stimuvax^®^ is a therapeutic vaccine indicated for certain types of cancer expressing tumor-specific antigens. This cancer vaccine is based on an antigenic lipopeptide, tecemotide, that targets mucin-1, which is overexpressed in various tumors, including breast, prostate, non-small-cell lung, and colorectal cancer. Tecemotide is encapsulated within a liposomal formulation composed of cholesterol, dimyristoylphosphatidylglycerol, and dipalmitoylphosphatidylcholine. In fact, it was the first liposomal cancer vaccine to enter phase III clinical trials (namely, the INSPIRE (NCT01015443), STRIDE (NCT00925548), START (NCT00409188), and START2 (NCT02049151) trials) [43]. The phase III clinical trial START aimed to determine whether Stimuvax^®^ in addition to best supportive care was effective in unresectable non-small-cell lung cancer compared with best supportive care alone. However, Stimuvax^®^ failed to meet its primary endpoint in terms of overall survival [121].

Concerning chemotherapy, there are several formulations under phase III clinical trials. Four are formulated as liposomes, two are formulated as micelles, one is formulated as polymeric nanoparticles, and one is formulated as a polymer conjugate.

Two distinct formulations have been developed to reduce the systemic toxicity of cisplatin. SPI-77 are 110-nm-sized stealth liposomes containing cisplatin and composed of hydrogenated soy phosphatidylcholine, cholesterol, and methoxy-PEG-phospho-ethanolamine (in a 51:44:5 molar ratio) [122]. SPI-77 has completed a phase III clinical trial (NCT00416507) that evaluated the overall survival achieved with gemcitabine, fluorouracil, liposomal cisplatin, and radiation in comparison with gemcitabine alone in nonmetastatic nonresectable pancreatic cancer. Nevertheless, the results show that this intensive induction schedule was more toxic and less effective than gemcitabine alone [123]. Another cisplatin formulation that has entered phase III clinical trials is Nanoplatin (NC-6004). This formulation encapsulates cisplatin into 30-nm-sized polymeric micelles of polyethylene glycol–poly(glutamic acid) block copolymers [124]. Nanoplatin has recently completed a phase III clinical trial (NCT02043288) that aimed to evaluate the impact of the addition of Nanoplatin to gemcitabine for the treatment of advanced or metastatic pancreatic cancer in Asian countries. The results are not yet available.

Moreover, the first antibody-targeted liposomal formulation (MM-302) has entered phase III clinical trials. MM-302 consists of 75–110-nm-sized doxorubicin-loaded stealth liposomes conjugated to a monoclonal antibody against the human epidermal growth factor receptor 2 (HER-2) under the assumption that it could enhance the delivery of doxorubicin to tumors overexpressing the HER-2 receptor. The HERMIONE phase II/III trial (NCT02213744) evaluated the efficacy of the addition of MM-302 to trastuzumab for metastatic HER2-positive breast cancer. Unfortunately, the study was prematurely terminated because it was found to not confer any benefit when compared with the control [44]. Another novel doxorubicin liposomal formulation has also progressed to phase III clinical trials. ThermoDox^®^ is the first thermoresponsive liposome to reach clinical development that quickly and efficiently releases doxorubicin in response to mild increases in temperature. These liposomes have a mean diameter of 100 nm and are composed of 1,2-dihexadecanoyl-sn-glycero-3-phosphocholine, myristoylstearoyl phosphatidylcholine, and 1,2-distearoyl-sn-glycero-3-phosphoethanolamine-*N*-[amino PEG-2000] (in a 86:10:4 molar ratio). The phase transition temperature of these phospholipids (around 40 °C) enables doxorubicin to be retained within the liposomes while circulating in the bloodstream and released following membrane permeabilization upon moderate local hyperthermia. To this end, ThermoDox^®^ is administered in combination with radio-frequency ablation (RFA), microwave hyperthermia, or high-intensity focused ultrasound [125,126]. Temperatures between 39 and 42 °C trigger the release of doxorubicin from liposomes and help to produce a high drug concentration of the drug in the tumor. As a result, ThermoDox is deemed to be suitable for the treatment of HCC since the treatment of this malignancy routinely includes the use of RFA. ThermoDox^®^ has completed two phase III trials (namely, NCT00617981 and the OPTIMA study NCT021112656) for the treatment of nonresectable HCC in conjunction with RFA. While the first study did not show any statistically significant increase in progression-free survival, the results from the OPTIMA study are still pending. Livatag^®^ is an alternative doxorubicin formulation based on the Transdrug™ technology, which relies on the use of 300-nm-sized poly isohexylcyanoacrylate nanoparticles. The efficacy of Livatag for the treatment of advanced HCC after failure of or intolerance to sorafenib was determined in the ReLive phase III clinical trial (NCT01655693). Unfortunately, Livatag did not improve the overall survival of patients with hepatocellular carcinoma in whom a previous sorafenib treatment had failed [127].

Furthermore, Endotag-1^®^ is a paclitaxel liposomal formulation that is currently under two phase III trials. Endotag-1 consists of 180–200-nm-sized liposomes composed of the cationic dioleoyl-oxy-propyl-trimethylammonium and 1,2-dioleoyl-sn-glycero-3-phosphocholin (in a 50:50 molar ratio) under the assumption that cationic liposomes interact to a greater extent with the negatively charged endothelial cells from the tumor’s neovasculature [49,128]. The NCT03126435 trial aims to evaluate the efficacy of the addition of Endotag-1 to gemcitabine for the treatment of locally advanced/metastatic pancreatic cancer after FOLFIRINOX failure. FOLFIRINOX is a combination chemotherapy regimen that includes folinic acid, fluorouracil, irinotecan hydrochloride, and oxaliplatin used to treat pancreatic cancer. The NCT03002103 trial aims to test the efficacy of the addition of Endotag-1 to a combination of paclitaxel and gemcitabine as a first-line therapy for visceral metastatic triple-negative breast cancer. Both phase III trials are currently recruiting patients. NK105 constitutes an alternative paclitaxel formulation based on 85-nm-sized polymeric micelles of amphiphilic PEG-poly(aspartic acid) block copolymers [129]. A phase III clinical trial (NCT01644890) evaluating the efficacy of NK105 for metastatic or recurrent breast cancer failed to meet the primary endpoint in terms of progression-free survival [130].

Finally, polymer conjugates have also been applied to chemotherapeutics. Onzeald^®^ is a four-arm PEG conjugate of irinotecan conjugated via a biodegradable, cleavable, ester-based linker that provides an extended release of irinotecan. Onzeald has completed two phase III clinical trials. The BEACON study (NCT01492101) compared the overall survival of patients who received Onzeald given intravenously once every three weeks to that of patients who received a treatment a physician selected from a list of seven single-agent intravenous therapies. The results from this trial did not demonstrate an improvement in overall survival for etirinotecan pegol compared with the physician selected treatment in patients with heavily pretreated advanced breast cancer [131]. On 20 July 2017, the Committee for Medicinal Products for Human Use recommended that marketing authorization for Onzeald be refused on the grounds that the claim of effectiveness relied on data from a subgroup of patients from the BEACON study that were not sufficient to prove the efficacy of Onzeald. Therefore, the ATTAIN study (NCT02915744) was subsequently launched to focus on the subset of patients that seemed to obtain a survival benefit from Onzeald in the BEACON study. The ATTAIN study compared Onzeald with a treatment of a physician’s choice in patients with breast cancer and a history of stable brain metastases that have previously been treated with an anthracycline, a taxane, and capecitabine [132].

Moreover, when discussing the latest achievements in novel formulations for chemotherapy, it is worth mentioning that BioPearl is the first formulation to be CE-marked in the new decade, having received the CE marking in April 2020 for chemoembolization of hepatocellular carcinoma. BioPearl’s approval hit a milestone as it contains the first biodegradable drug-eluting microspheres for transarterial chemoembolization. Although no further information is available at present, the postmarket follow-up study BioPearl-FIRST (NCT04231929) has been launched to confirm the safety and efficacy of BioPearl microspheres loaded with doxorubicin in the treatment of patients with unresectable hepatocellular carcinoma. The BIOPEARL-FIRST study is not yet recruiting patients.

However, whereas considerable success has been achieved in the extended-release delivery of short half-life peptide-based therapies, the targeting efficiency of delivery systems has solely relied on passive targeting, which has proven to be limited. In this context, researchers are paving the way for three promising strategies, which are currently in preclinical stages of evaluation: active targeting, stimuli-responsive systems, and the design of multifunctional systems [133,134]. Considerable effort is being made to maximize the accumulation of peptides, chemotherapeutics, and radiotherapeutics at the site of interest by these methods [135].

Active targeting consists of the attachment of different targeting moieties to the surface of nanomedicines, which, afterwards, will interact specifically with membrane receptors that are either uniquely expressed or overexpressed on the tumor cells [136,137]. Frequently used targeting ligands include small molecules [138], tumor-homing peptides [139], proteins [140], antibodies [141], and aptamers [142]. The aforementioned MM-302 liposomes represent the single formulation exploiting active targeting that has progressed to phase III clinical trials for the treatment of cancer.

Stimuli-responsive systems have been rationally developed for the spatiotemporal control of drug release at the tumor site by internal or external stimuli. On the one hand, internal stimuli-responsive systems are usually designed with smart biomaterials able to trigger drug release in response to internal changes in the tumor microenvironment or cancer cells such as changes in pH, redox potential, and enzymatic activity [143,144]. First, there is a slight difference in pH between healthy tissues (7.4) and the extracellular environment of solid tumors (6.5–7.2) due to the increased production and slow exportation of lactate and CO_2_ [145,146]. Therefore, multiple anticancer drug delivery systems can be developed using polymers with ionizable groups that undergo conformational and/or solubility changes in response to pH variation [147]. Second, the redox potential difference between the oxidizing extracellular space and the reducing intracellular space can serve as a potential stimulus for the triggered release of therapeutic drugs as well. The formulation depends on the chemistry of the respective redox-responsive units, such as disulfide bonds [148]. Third, specific enzymes, such as hydrolases [149] or transferases [150], are overexpressed in certain types of cancer. Therefore, drug release can also be triggered at the tumor site by taking advantage of smart polymers used as carriers that are sensitive to cleavage by a particular enzyme [151].

On the other hand, external stimuli-responsive systems rely on the application of distinct stimuli, such as light, temperature, a magnetic field, or ultrasound using various external devices, offering multiple advantages: they could be used to target specific regions, as a switch to trigger drug release, and even enable different types of therapies. Regarding the first possibility, magnetic guidance is typically achieved by focusing an extracorporeal magnetic field on the biological target during the injection of a magnetically responsive nanocarrier (generally SPIONs). This concept has been demonstrated to have great potential in experimental cancer therapy because of the improved drug accumulation inside solid-tumor models [152,153]. There are three distinct options for exogenously triggering drug release from stimuli-responsive delivery systems. Firstly, thermosensitive systems can be designed to be stable at body temperature (up to 37 °C) and to undergo significant changes in their properties upon heating to above 40 °C to ultimately trigger cargo release in response to the slight temperature shift [154]. As an example, thermosensitive vesicles obtained good results in preclinical development [155]. ThermoDox^®^ provides an increase in drug release of between 25 and 5 times in comparison with free and standard liposomal doxorubicin, respectively, in tumors [156]. In order to provide the external temperature stimulus, heat is often induced by RFA, microwave hyperthermia, and high-intensity focused ultrasound. ThermoDox^®^ is the only thermoresponsive formulation to have reached phase III clinical trials. Secondly, photo-responsive systems have been engineered in the past few years to achieve on-demand drug release upon exposure to a specific wavelength [144]. For instance, stealth liposomes can be used as formulations to trigger doxorubicin release [157]. Thirdly, ultrasound represents an effective method for attaining spatiotemporal control over drug release at the target site, thus preventing harmful side effects on healthy tissues. Ultrasound can not only trigger the release of drugs from carriers but can also increase the permeability of biological barriers (cell membranes, the blood–brain barrier, etc.) through the formation of cavitation bubbles and increased temperature, resulting in enhanced drug diffusion. Microbubbles, liposomes, and emulsions are the most common systems that respond to ultrasound [158]. Finally, external-stimuli systems can be used in photothermal therapy (PTT) and photodynamic therapy (PDT). PTT is a minimally invasive method that utilizes light-absorbing agents to convert photon energy into thermal energy; thus, the generated hyperthermia can irreversibly damage cancer cells [159]. Specifically, gold nanomaterials, such as gold nanoparticles, are being used for PTT because of their great photothermal conversion ability and stability [160]. PDT is an approach in which photosensitizers under light exposure transfer the absorbed optical energy to surrounding oxygen molecules, generating reactive oxygen species that induce local tissue apoptosis and necrosis, killing cancer cells [161,162].

Given the extremely heterogeneous nature of cancer, each treatment could be most effective for certain patients at certain stages of the disease. In this context, theranostic nanomedicine, at the intersection between imaging and therapy, holds tremendous promise for the management of cancers. Theranostic carriers combine therapeutic and diagnostic functions within a single platform to enable monitoring of the tumor’s response to treatment and guide stimulus-responsive therapies and surgical resection to ultimately help tailor therapies to each patient’s individual needs [163]. Many nanomaterials, including gold nanoparticles, quantum dots, carbon nanotubes, and mesoporous silica nanoparticles, possess intrinsic therapeutic/diagnostic properties, although SPIONs are currently being the most extensively studied as theranostic candidates due to the superiority of MRI in terms of providing high spatial resolution with no tissue-penetrating limitation [162]. Other techniques can also be used for imaging purposes, such as CT, which possesses high spatial resolution but has a poor soft-tissue contrast, and nuclear medicine imaging (positron emission tomography or single-photon emission computed tomography (SPECT)), which has high sensitivity but requires radioactive tracers that can put patients and operators at risk [164]. In this respect, the approval of DC Bead LUMI and QuiremSpheres represents a milestone as they are the first-ever theranostic systems to be marketed. Whereas DC Bead LUMI incorporates CT functions for visualization for efficient chemoembolization, QuiremSpheres can be imaged both by SPECT/CT and MRI for efficient SIRT.

In addition, carriers are also being engineered to combine the attachment of active moieties with multiple stimuli-responsive functions, facilitating multistage drug delivery as well as achieving higher specificity and efficacy. These aspects would lead to an improvement in disease management and minimize the risks [165]. For instance, nanocarriers that respond to both intracellular pH and intracellular redox potential have been developed for the purpose of enhancing intracellular drug delivery [166]. In another study, a dual hypoxia-responsive drug delivery system was developed in order to enable PDT-induced drug release and drug activation mediated via PDT-induced hypoxia [167].

## 6. Successes, Failures, and Lessons Learned

**Figure 5:**
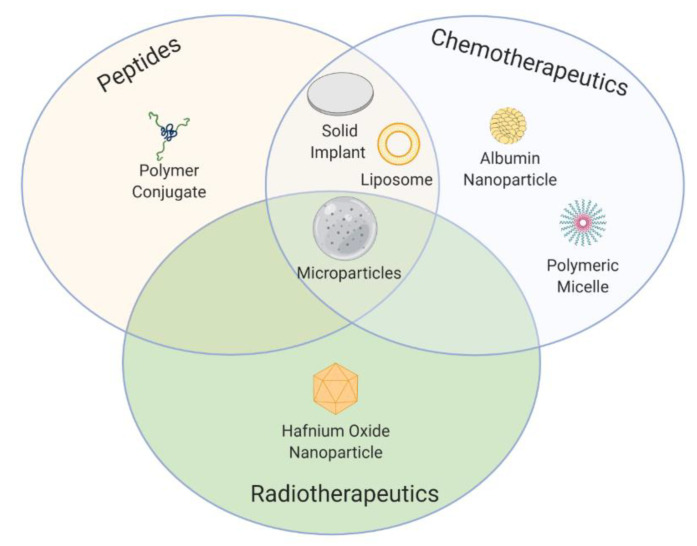
Scheme of approved formulation types along the past three decades for cancer treatment classified by the type of therapy.

Over the past few decades, a wide range of innovative formulation strategies and pioneering technologies have reached the market (Figure 5) and entered into clinical trials as a result of the extensive efforts made in preclinical and clinical stages.

Concerning peptide-based therapy, the main aim pursued with novel formulation strategies has been to overcome the short elimination half-life of peptides. In this regard, the introduction into the market of the first microspheres and implants using biodegradable polymers in the 1990s for prolonged peptide release has enabled the administration of some peptides for cancer treatment that were not clinically available (namely, goserelin) and a reduction in the frequency of administrations, improving thereby patient compliance, of some other peptides that were previously available in daily injectable formulations (such as buserelin, triptorelin, leuprolide, and octreotide). The subsequent authorization of Vantas^®^ and Viadur^®^ in the 2000s, which extended peptide release over a year, further reduced the frequency of administration in comparison with the previous 3–4-months’ implants [168]. Unfortunately, Viadur^®^ was discontinued due to diminished market demand and growing manufacturing costs. However, during the 2010s the innovation in microspheres and implant-based formulation technologies for peptide delivery has been abruptly reduced. It could be due to a change in tendency as 75% of all nonmarketed formulations that have entered phase III clinical trials for peptide-based therapy are polymer conjugates. However, these data should be handled cautiously, given that these phase III trials had a negative outcome, or their results are still pending.

Hormone-dependent cancers have been favored to the greatest extent by prolonged-release systems of peptide-based therapies because these cancers require continuous administration over long periods. In fact, 75% of all formulations authorized for peptide-based therapy are designed to treat hormone-dependent cancers, of which prostate cancer is the most treated [169]. This can be seen as a major limitation of peptide-based therapy, as these formulations are restricted to hormone-dependent cancers. This trend, however, is reversed in phase III clinical trials, as 50% of the nonmarketed formulations for peptide-based therapy are being tested for the treatment of pancreatic cancer, suggesting that the interest in using peptide-based formulations to treat other cancers is increasing. Nevertheless, the translational potential of peptide-based therapy for other types of cancers remains to be fully demonstrated, as phase III trials with peptide-based therapy for pancreatic cancer failed to meet their primary outcomes.

Concerning chemotherapeutics, the main aim pursued with novel formulation strategies has been to improve their nonspecific distribution. In this regard, various carrier-based formulations incorporating chemotherapeutics have received marketing authorization in order to reduce their toxicity and improve their therapeutic index. Ever since Doxil^®^ was authorized in the 1990s as the first liposome formulation entrapping doxorubicin, liposomes have been trending upward in the market [170]. Many of them have introduced innovations such as Vyxeos^®^ with the incorporation of two chemotherapeutics within the same formulation. In this regard, Vyxeos^®^ may well set the foundation for future approvals exploring alternative drug combinations. In addition to their high biocompatibility, one reason why liposomes have been in the limelight over the past three decades may be the good results obtained by reducing the side effects of chemotherapy in the clinic. The reduction in the neurotoxicity using vincristine and the reduction in the cardiotoxicity of doxorubicin after encapsulation within liposomes are two representative examples [46,89,171] Other nanomedicines apart from liposomes have received marketing authorization for chemotherapy. This is the case of Abraxane^®^ in the 2000s, the first nanoparticle-based formulation using albumin as a carrier and, more recently, the first micelle-based formulation entrapping paclitaxel in 2018 under the tradename Apealea^®^. Alternatively, during the 2000s and 2010s, microspheres containing chemotherapeutics were authorized for chemoembolization following local administration. Further improvements in these microspheres resulted in the first commercially available microsphere that combines chemotherapy with imaging function (DC Bead LUMI). Despite current research efforts, the combination of imaging and therapeutic functions remains to be achieved with nanomedicines at the clinical level.

However, the improvement of the therapeutic index of chemotherapy agents with these formulations is more related to an increase in the safety profile of the formulation than to an increase in therapeutic efficacy, which remains a challenge for the decades to come. Although the use of nanocarriers exploiting passive targeting through the EPR effect was thought to substantially improve the nonspecific distribution of chemotherapeutics, tumor accumulation remains a major hurdle as the extent of the EPR effect varies widely among patients and tumor type [170,172]. In fact, despite the wide range of preclinical studies on the pegylation strategy, only a few authorized formulations are actually stealth liposomes. Hence, it is worth noting that there currently seems to be a dilemma about whether or not the use of PEG is worthwhile [173]. The polymer chains that prevent the recognition of opsonins and subsequent phagocytosis by the RES may also prevent liposomes from being internalized by target cells and the PEG layer may even contribute to developing palmar–plantar erythrodysesthesia [174]. Later, researchers started to focus on the functionalization of the carrier with targeting moieties to exploit active targeting as an alternative to passive targeting. Nevertheless, the reality is that none of the formulations authorized during the past three decades are based on this strategy. Despite the wide range of preclinical studies on active targeting during the 2010s, its immediate clinical translation remains uncertain, as the phase III trial with the first antibody-targeted liposomal formulation (MM-302) was prematurely terminated. It seems that the high variability in receptor expression between tumors is directly related to the failure of active targeting [98,175]. Alternatively, microspheres for chemoembolization increase the selectivity of the chemotherapeutics following intra-arterial administration, but at the cost of causing discomfort to the patient, as this type of administration is a complex local invasive procedure that requires trained professionals to perform it.

Moreover, some other difficulties encountered with clinical translation are related to manufacturing issues. Platforms that require complex and/or laborious synthesis procedures generally have a limited potential for clinical translation, as they can be difficult to scale up. For example, of the 16 technologically modified formulations entrapping chemotherapeutics that have reached the market, DaunoXome^®^ and DepoCyt^®^ have been discontinued due to manufacturing issues.

Of all the cancers treated with chemotherapeutic agents, the most common indication is hepatocellular carcinoma (37.5%), which accounts for >80% of primary liver cancers worldwide [176], followed by breast cancer (19%), mostly as second-line and hematological malignancies (19%). Of the nonmarketed formulations for the delivery of chemotherapeutics that are currently being evaluated in clinical trials, breast cancer represents the most frequent indication (50%), followed by adenocarcinoma of the pancreas (37.5%). However, as it also occurred with peptide-based therapy, future trends should be analyzed with caution, given that six of these phase III trials with chemotherapeutic formulations had a negative outcome

Analogously to chemotherapeutics, concerning radiotherapeutics, the main aim pursued with novel formulation strategies has been to improve their nonspecific distribution in order to deliver the maximum amount of radiation to the tumor with minor radiation-induced damage. Microspheres containing radiotherapeutics have been authorized for radioembolization. Based on the encouraging results obtained with selective local procedures such as TACE and the lack of efficient targeting strategies observed following systemic administration, all microspheres for radiotherapy approved in the past three decades are given following intra-arterial administration. Further improvements in these microspheres resulted in the first commercially available microsphere that combines radiotherapy with imaging function (QuiremSpheres^®^). Alternatively, in 2019 a new class of radiation-enhancing nanoparticles formulated in crystalline HfO2 were authorized in Europe, being the first generation of radio enhancers intratumorally administered available for the treatment of solid tumors. The most common indication for formulations encapsulating radiotherapeutics is hepatocellular carcinoma (75%) followed by sarcoma (25%). As long as targeting efficiency following systemic administration is not enhanced, this type of therapy will only be suitable for accessible cancer types in which local administration is enabled. No phase III clinical trial with nonmarketed formulations of radiotherapeutics has been active in the last five years.

**Figure 6:**
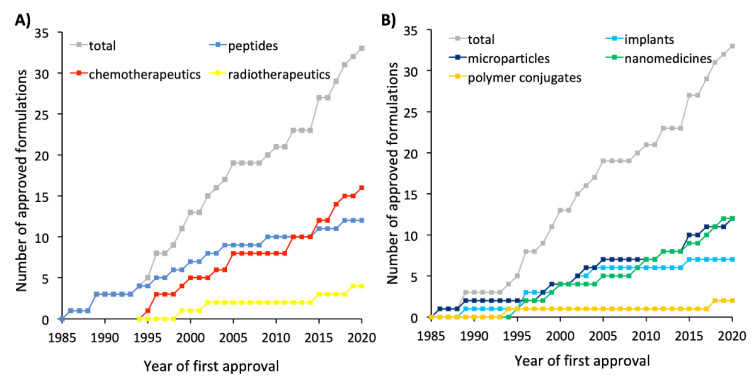
(**A**) Evolution of the approved formulations for cancer classified by the type of therapy (peptide-based therapy, chemotherapy, or radiotherapy). (**B**) Evolution of the approved formulations for cancer classified by the type of formulation (implants, microparticles, nanomedicines, or polymer conjugates). The term “nanomedicines” encompasses liposomes, peptide-based nanoparticles, micelles, and inorganic nanoparticles. The year of first approval is reported for formulations approved by both the FDA and EMA.

Figure 1, Figure 2 and Figure 3 reflect how the formulations for cancer treatment have evolved depending on the type of therapy and the type of technology used. According to Figure 1a, Figure 2a and Figure 3a, the formulations for peptide-based therapy have gone from representing 55% of the total number of new formulations approved during the 1990s to account for only 17% of the formulations approved in the 2010s. A clear parallel can be drawn between this decrease and the increase in the number of novel formulations for the delivery of chemotherapeutics, which has risen from 36% of the formulations approved during the 1990s to 58% of all formulations approved during the 2010s. The percentage of radiotherapeutics has slowly increased as well. Figure 6a shows the evolution of the approved formulations for the three types of cancer therapy to be compared. Between 2010 and 2015 the number of approved formulations for chemotherapy surpassed the number of peptide-based formulations that received authorization. Apart from the types of therapy, it is important to mention how the types of authorized formulations have evolved over time. As shown in Figure 6b, concerning the type of formulation used, both microcarriers and nanocarriers lead the marketed formulations (36.5%), whereas solid implants and polymer conjugates are lagging behind (21% and 6%, respectively). Figure 1b, Figure 2b and Figure 3b illustrate how liposomes and microspheres have been in the limelight over the past three decades while implants have been trending downwards since the 2000s.

Regarding the new formulation technologies that are expected to be approved in the future, as mentioned in Section 5, active targeting, stimuli-responsive systems, and multifunctional systems are setting trends at the preclinical level. However, according to Table 4, only MM-302, an actively targeted liposomal formulation, and ThermoDox^®^, a thermosensitive liposomal formulation, have reached phase III clinical trials. Hence, clinical translation of these sophisticated systems is not expected to occur in the short term. Most of the formulations that have reached phase III clinical trials are liposomes for chemotherapeutics and polymer conjugates for peptides (Figure 4). As a novelty, Stimuvax^®^, a vaccine for non-small-cell lung cancer, has completed a phase III clinical trial.

Regulatory agencies play an important role in the approval of novel formulations. Even though some of the products reviewed herein are not approved either in Europe or in the United States, 42.4% of all formulations are approved by both regulatory agencies (although approval does not necessarily occur concomitantly; e.g., Oncaspar^®^ was approved by the EMA 22 years after being approved by the FDA). Some formulations, including Suprefact^®^, Mepact^®^, Myocet^®^, DC Bead^®^, HepaSphere^®^, Leptoprol^®^, Apealea^®^, LifePearl^®^, Embozene Tandem^®^, DC Bead LUMI^®^, QuiremSpheres^®^, Hensify^®^, NanoTherm^®^, and BioPearl^®^, are authorized for use only in Europe, and Viadur^®^, Vantas^®^, Asparlas^®^, and Marqibo^®^ only in the United States. Much of this percentage mismatch is attributable to the DEM-TACE microspheres, which have been approved as medical devices in Europe and the United States, but the drug-loading feature has yet to be approved by the FDA [177]. Overall, drug products represent 66.7% of the total number of approved formulations for cancer therapy, whereas medical devices represent the remaining 33.3%. However, these data are not uniform among the distinct types of cancer therapy; whereas 100% of the products approved for peptide-based therapy are drug products, the trend is reversed in the case of radiotherapy, where medical devices account for 100% of the approved formulations.

In conclusion, despite the lessons learned during these three decades that have allowed us to acquire a profound technological and pharmacological understanding of the development of novel formulations that have led to important advances in the treatment of cancer, there is still a long way to go to continue to improve the field of cancer therapy.

## Figures and Tables

**Table 1:** Formulations for cancer therapy authorized between 1986 and 1999 grouped by the type of therapy. IM: intramuscular; SC: subcutaneous; IV: intravenous; IC: intracranial; ITh: intrathecal; IA: intra-arterial. *: drug release extends over 3 weeks, but the implant is intended for a single administration during surgery.

Type of Therapy	Formulation Type	Mechanism of Action	Drug Substance	Trade Name	Administration Route	Dosing Frequency	Indications	First Approval	Current Status
Peptide-based therapy	Microspheres	Inhibition of gonadotropin secretion	Triptorelin acetate	Decapeptyl	IM	1–6 months	Prostate cancer	1986	Active
	Microspheres	Inhibition of gonadotropin secretion	Leuprolide acetate	Lupron Depot	IM	1–6 months	Prostate cancer	1989	Active
	Solid implant	Inhibition of gonadotropin secretion	Goserelin acetate	Zoladex	SC	1–3 months	Prostate and breast cancer	1989	Active
	Polymer conjugate	l-asparagine depletion	Pegaspargase	Oncaspar	IV, IM	2 weeks	Lymphoblastic leukemia	1994	Active
	Solid implant	Inhibition of gonadotropin secretion	Buserelin acetate	Suprefact Depot	SC	2–3 months	Prostate cancer	1996	Active
	Microspheres	Inhibition of secretion of peptides from the endocrine gastrointestinal system	Octreotide acetate	Sandostatin LAR	IM	1 month	Neuroendocrine tumors	1998	Active
Chemotherapy	Liposomes	Topoisomerase-II inhibition, DNA intercalation	Doxorubicin	Doxil	IV	3–4 weeks	AIDS-related Kaposi’s sarcoma, ovarian, and breast neoplasms, multiple myeloma	1995	Active
	Liposomes	Topoisomerase-II inhibition, DNA intercalation	Daunorubicin	Daunoxome	IV	2 weeks	AIDS-related Kaposi’s sarcoma	1996	Discontinued
	Solid implant	DNA alkylation	Carmustine	Gliadel	IC	3 weeks *	Malignant glioma	1996	Active
	Liposomes	DNA polymerase inhibition	Cytarabine	DepoCyt	ITh	4 weeks	Lymphomatous meningitis	1999	Discontinued
Radiotherapy	Microspheres	Beta particle emission	Yttrium-90	Theraspheres	IA	-	Hepatocellular carcinoma	1999	Active

**Table 2:** Formulations for cancer therapy authorized between 2000 and 2009 grouped by the type of therapy. SC: subcutaneous; IV: intravenous; IA: intra-arterial. * Conformité Européenne (CE)-marked for loading with doxorubicin or irinotecan.

Type of Therapy	Formulation Type	Mechanism of Action	Drug Substance	Trade Name	Administration Route	Dosing Frequency	Indications	First Approval	Current Status
Peptide-based therapy	Solid implant	Inhibition of gonadotropin secretion	Leuprolide acetate	Viadur	SC	12 months	Prostate cancer	2000	Discontinued
	In-situ-forming implants	Inhibition of gonadotropin secretion	Leuprolide acetate	Eligard	SC	1–6 months	Prostate cancer	2002	Active
	Solid implant	Inhibition of gonadotropin secretion	Histrelin acetate	Vantas	SC	12 months	Prostate cancer	2004	Active
	Liposomes	Immunomodulation	Mifamurtide	Mepact	IV	1/2–1 week	Osteosarcoma	2009	Active
Chemotherapy	Liposomes	Topoisomerase-II inhibition, DNA intercalation	Doxorubicin	Myocet	IV	3 weeks	Metastatic breast cancer	2000	Active
	Microspheres	-	Various *	DC Bead	IA	-	Hepatocellular carcinoma	2003	Active
	Protein-based nanoparticles	Microtubule inhibition	Paclitaxel	Abraxane	IV	1–3 weeks	Breast neoplasms, pancreatic neoplasms, non-small-cell lung cancer	2005	Active
	Microspheres	-	Various *	Hepasphere	IA	-	Hepatocellular carcinoma	2005	Active
Radiotherapy	Microspheres	Beta particle emission	Yttrium-90	SIR-Spheres	IA	-	Metastatic liver tumors	2002	Active

**Table 3:** Formulations for cancer therapy authorized between 2010 and 2019 grouped by the type of therapy. SC: subcutaneous; IV: intravenous; IA: intra-arterial. IT: intratumoral; * CE-marked for loading of doxorubicin or irinotecan, ** CE-marked for loading with doxorubicin, irinotecan, idarubicin, or epirubicin.

Type of Therapy	Formulation Type	Mechanism of Action	Drug Substance	Trade Name	Administration Route	Dosing Frequency	Indications	First Approval	Current Status
Peptide-based therapy	Solid implant	Inhibition of gonadotropin secretion	Leuprolide acetate	Leptoprol	SC	3 months	Prostate cancer	2015	Active
	Polymer conjugate	l-asparaginase depletion	Calaspargase pegol	Asparlas	IV, IM	3 weeks	Acute lymphoblastic leukemia	2018	Active
Chemotherapy	Liposomes	Microtubule inhibition	Vincristine	Marqibo	IV	1 week	Acute lymphoblastic leukemia	2012	Active
	Microspheres	-	Various *	Embozene TANDEM	IA	-	Hepatocellular carcinoma	2012	Active
	Liposomes	Topoisomerase I inhibition	Irinotecan	Onivyde	IV	2 weeks	Metastatic adenocarcinoma of the pancreas	2015	Active
	Microspheres	-	Various **	Life Pearl	IA	-	Hepatocellular carcinoma	2015	Active
	Liposomes	DNA polymerase inhibition + topoisomerase-II inhibition, DNA intercalation	Cytarabine + daunorubicin	Vyxeos	IV	2 days	Acute myeloid leukemia	2017	Active
	Microspheres	-	Various *	DC Bead LUMI	IA	-	Hepatocellular carcinoma	2017	Active
	Micelles	Microtubule inhibition	Paclitaxel	Apealea	IV	3 weeks	Ovarian cancer, primary peritoneal cancer, fallopian tube cancer	2018	Active
Radiotherapy	Microspheres	Beta particle emission	Holmium-166	QuiremSpheres	IA	-	Hepatocellular carcinoma	2015	Active
	Inorganic nanoparticles	Radio enhancer	Hafnium oxide	Hensify	IT	-	Soft-tissue sarcoma	2019	Active
Other	Inorganic nanoparticles	Hyperthermia	Iron oxide	NanoTherm	IT	-	Glioblastoma	2010	Active

**Table 4:** Novel nonmarketed formulations for cancer therapy that have entered phase III clinical trials. SC: subcutaneous, IM: intramuscular, IV: intravenous, IL-10: interleukin 10.

Type of Therapy	Formulation Type	Mechanism of Action	Drug Substance	Trade Name	Administration Route	Indications	Clinical Trial Number	Current Status
Peptide-based therapy	Liposomes	Immunomodulation	Tecemotide	Stimuvax	SC	Non-small-cell lung cancer	NCT00409188	Completed
	Polymer conjugate	Arginine depletion	Pegargiminase	ADI-PEG 20	IM	Mesothelioma	NCT02709512	Recruiting
	Polymer conjugate	Hyaluronan degradation	Pegvorhyaluronidase alfa	PEGPH20	IV	Pancreatic cancer	NCT02715804	Terminated
	Polymer conjugate	Immunomodulation	Pegylated IL-10	Pegilodecakin	SC	Pancreatic cancer	NCT02923921	Completed
Chemotherapy	Liposomes	Topoisomerase-II inhibition, DNA intercalation	Doxorubicin	MM-302	IV	Breast cancer	NCT02213744	Terminated
	Liposomes	Topoisomerase-II inhibition, DNA intercalation	Doxorubicin	Thermodox	IV	Hepatocellular carcinoma	NCT00617981	Completed
							NCT02112656	Completed
	Liposomes	Microtubule inhibition	Paclitaxel	Endotag 1	IV	Breast cancer	NCT03002103	Recruiting
						Pancreatic cancer	NCT03126435	Recruiting
	Liposomes	DNA alkylation	Cisplatin	SPI-77	IV	Pancreatic cancer	NCT00416507	Completed
	Polymer conjugate	Topoisomerase I inhibition	Etirinotecan pegol	Onzeald	IV	Breast cancer	NCT01492101	Completed
							NCT02915744	Completed
	Micelles	DNA alkylation	Cisplatin	Nanoplatin	IV	Pancreatic cancer	NCT02043288	Completed
	Micelles	Microtubule inhibition	Paclitaxel	NK105	IV	Breast cancer	NCT01644890	Completed
	Polymeric nanoparticles	Topoisomerase-II inhibition, DNA intercalation	Doxorubicin	Livatag	IV	Hepatocellular carcinoma	NCT01655693	Completed

## Abbreviations

ALL	acute lymphoblastic leukemia
AML	acute myeloid leukemia
CE	Conformité Européenne
DEM	drug-eluting microspheres
DEM-TACE	drug-eluting beads in transarterial chemoembolization
EPR	enhance permeability and retention effect
GnRH	gonadotropin-releasing hormone
HCC	hepatocellular carcinoma
HER-2	human epidermal growth factor receptor 2
HfO2	hafnium oxide
IA	intra-arterial
IC	intracranial
IL-10	interleukin 10
IM	intramuscular
IT	intratumoral
ITh	intrathecal
IV	intravenous
MNP	magnetic nanoparticles
mPEG	monomethoxy-polyethylene glycol
MRI	magnetic resonance imaging
Nab	nanoparticle albumin-bound
PDT	photodynamic therapy
PEG	polyethylene glycol
PLA	poly(lactic acid)
PLGA	lactic-co-glycolic acid
PTT	photothermal therapy
RES	reticuloendothelial system
RFA	radio-frequency ablation
SC	subcutaneous
SIRT	selective internal radiation therapy
SPECT	single-photon emission computed tomography
SPECT/CT	single-photon emission computed tomography/computed tomography
SPIONs	superparamagnetic iron oxide nanoparticles
TACE	transarterial chemoembolization
TTP	time to progression

## References

[1] Bray F., Ferlay J., Soerjomataram I., Siegel R.L., Torre L.A., Jemal A. (2018). Global cancer statistics 2018: GLOBOCAN estimates of incidence and mortality worldwide for 36 cancers in 185 countries. CA Cancer J. Clin..

[2] DeVita V.T., Chu E. (2008). A History of Cancer Chemotherapy. Cancer Res..

[3] Arruebo M., Vilaboa N., Sáez-Gutierrez B., Lambea J., Tres A., Valladares M., González-Fernández Á. (2011). Assessment of the evolution of cancer treatment therapies. Cancers.

[4] Miller K.D., Nogueira L., Mariotto A.B., Rowland J.H., Yabroff K.R., Alfano C.M., Jemal A., Kramer J.L., Siegel R.L. (2019). Cancer treatment and survivorship statistics, 2019. CA Cancer J. Clin..

[5] Xiao Y.F., Jie M.M., Li B.S., Hu C.J., Xie R., Tang B., Yang S.M. (2015). Peptide-Based Treatment: A Promising Cancer Therapy. J. Immunol. Res..

[6] Barata P., Layton J., Lewis B., Sartor O. (2020). Next Generation of Androgen Deprivation Therapy Combined With Radiotherapy for N0 M0 Prostate Cancer. Cancer J..

[7] Thundimadathil J. (2012). Cancer Treatment Using Peptides: Current Therapies and Future Prospects. J. Amino Acids.

[8] Zafar S., Beg S., Panda S.K., Rahman M., Alharbi K.S., Jain G.K., Ahmad F.J. (2019). Novel therapeutic interventions in cancer treatment using protein and peptide-based targeted smart systems. Semin. Cancer Biol..

[9] Marqus S., Pirogova E., Piva T.J. (2017). Evaluation of the use of therapeutic peptides for cancer treatment. J. Biomed. Sci..

[10] Sagnella S.M., McCarroll J.A., Kavallaris M. (2014). Drug delivery: Beyond active tumour targeting. Nanomed. Nanotechnol. Biol. Med..

[11] Kishan A.U., Cook R.R., Ciezki J.P., Ross A.E., Pomerantz M.M., Nguyen P.L., Shaikh T., Tran P.T., Sandler K.A., Stock R.G. (2018). Radical prostatectomy, external beam radiotherapy, or external beam radiotherapy with brachytherapy boost and disease progression and mortality in patients with gleason score 9-10 prostate cancer. JAMA J. Am. Med. Assoc..

[12] De Ruysscher D., Niedermann G., Burnet N.G., Siva S., Lee A.W.M., Hegi-Johnson F. (2019). Radiotherapy toxicity. Nat. Rev. Dis. Prim..

[13] Faisant N., Akiki J., Siepmann F., Benoit J.P., Siepmann J. (2006). Effects of the type of release medium on drug release from PLGA-based microparticles: Experiment and theory. Int. J. Pharm..

[14] Kempe S., Mäder K. (2012). In situ forming implants—An attractive formulation principle for parenteral depot formulations. J. Control. Release.

[15] Hrkach J., Langer R. (2020). From micro to nano: Evolution and impact of drug delivery in treating disease. Drug Deliv. Transl. Res..

[16] Kalyane D., Raval N., Maheshwari R., Tambe V., Kalia K., Tekade R.K. (2019). Employment of enhanced permeability and retention effect (EPR): Nanoparticle-based precision tools for targeting of therapeutic and diagnostic agent in cancer. Mater. Sci. Eng. C.

[17] Van der Meel R., Vehmeijer L.J.C., Kok R.J., Storm G., van Gaal E.V.B. (2013). Ligand-targeted particulate nanomedicines undergoing clinical evaluation: Current status. Adv. Drug Deliv. Rev..

[18] Aparicio-Blanco J., Sanz-Arriazu L., Lorenzoni R., Blanco-Prieto M.J. (2020). Glioblastoma chemotherapeutic agents used in the clinical setting and in clinical trials: Nanomedicine approaches to improve their efficacy. Int. J. Pharm..

[19] Chew S.A., Danti S. (2017). Biomaterial-Based Implantable Devices for Cancer Therapy. Adv. Healthc. Mater..

[20] Bazak R., Houri M., El Achy S., Hussein W., Refaat T. (2014). Passive targeting of nanoparticles to cancer: A comprehensive review of the literature. Mol. Clin. Oncol..

[21] Taha M.S., Padmakumar S., Singh A., Amiji M.M. (2020). Critical quality attributes in the development of therapeutic nanomedicines toward clinical translation. Drug Deliv. Transl. Res..

[22] Dhaliwal A., Zheng G. (2019). Improving accessibility of EPR-insensitive tumor phenotypes using EPR-adaptive strategies: Designing a new perspective in nanomedicine delivery. Theranostics.

[23] Jasim A., Abdelghany S., Greish K. (2017). Current Update on the Role of Enhanced Permeability and Retention Effect in Cancer Nanomedicine. Nanotechnology-Based Approaches for Targeting and Delivery of Drugs and Genes.

[24] Zhang C., Wu L., Tao A., Bera H., Tang X., Cun D., Yang M. (2020). Formulation and in vitro characterization of long-acting PLGA injectable microspheres encapsulating a peptide analog of LHRH. J. Mater. Sci. Technol..

[25] Shi N.-Q., Zhou J., Walker J., Li L., Hong J.K.Y., Olsen K.F., Tang J., Ackermann R., Wang Y., Qin B. (2020). Microencapsulation of luteinizing hormone-releasing hormone agonist in poly (lactic-co-glycolic acid) microspheres by spray-drying. J. Control. Release.

[26] Jain A., Kunduru K.R., Basu A., Mizrahi B., Domb A.J., Khan W. (2016). Injectable formulations of poly(lactic acid) and its copolymers in clinical use. Adv. Drug Deliv. Rev..

[27] Ye M., Kim S., Park K. (2010). Issues in long-term protein delivery using biodegradable microparticles. J. Control. Release.

[28] Pandita D., Kumar S., Lather V. (2015). Hybrid poly(lactic-co-glycolic acid) nanoparticles: Design and delivery prospectives. Drug Discov. Today.

[29] Park K., Jung G.Y., Kim M.K., Park M.S., Shin Y.K., Hwang J.K., Yuk S.H. (2012). Triptorelin acetate-loaded poly(lactide-co-glycolide) (PLGA) microspheres for controlled drug delivery. Macromol. Res..

[30] Chen L., Ahmed A.M.Q., Deng Y., Cao D., Du H., Cui J., Lee B.-J., Cao Q. (2019). Novel triptorelin acetate-loaded microspheres prepared by a liquid/oil/oil method with high encapsulation efficiency and low initial burst release. J. Drug Deliv. Sci. Technol..

[31] Skidmore S., Hadar J., Garner J., Park H., Park K., Wang Y., Jiang X. (2019). (Jeff) Complex sameness: Separation of mixed poly(lactide-co-glycolide)s based on the lactide:glycolide ratio. J. Control. Release.

[32] Wechsel H.W., Zerbib M., Pagano F., Coptcoat M.J. (1996). Randomized Open Labelled Comparative Study of the Efficacy, Safety and Tolerability of Leuprorelin Acetate 1M and 3M Depot in Patients with Advanced Prostatic Cancer. Eur. Urol..

[33] Park K., Skidmore S., Hadar J., Garner J., Park H., Otte A., Soh B.K., Yoon G., Yu D., Yun Y. (2019). Injectable, long-acting PLGA formulations: Analyzing PLGA and understanding microparticle formation. J. Control. Release.

[34] Heo Y.-A., Syed Y.Y., Keam S.J. (2019). Pegaspargase: A Review in Acute Lymphoblastic Leukaemia. Drugs.

[35] Meneguetti G.P., Santos J.H.P.M., Obreque K.M.T., Barbosa C.M.V., Monteiro G., Farsky S.H.P., Marim de Oliveira A., Angeli C.B., Palmisano G., Ventura S.P.M. (2019). Novel site-specific PEGylated L-asparaginase. PLoS ONE.

[36] van der Meel R., Sulheim E., Shi Y., Kiessling F., Mulder W.J.M., Lammers T. (2019). Smart cancer nanomedicine. Nat. Nanotechnol..

[37] Parrish K., Sarkaria J., Elmquist W. (2015). Improving drug delivery to primary and metastatic brain tumors: Strategies to overcome the blood-brain barrier. Clin. Pharmacol. Ther..

[38] Shapira-Furman T., Serra R., Gorelick N., Doglioli M., Tagliaferri V., Cecia A., Peters M., Kumar A., Rottenberg Y., Langer R. (2019). Biodegradable wafers releasing Temozolomide and Carmustine for the treatment of brain cancer. J. Control. Release.

[39] Brem H., Piantadosi S., Burger P., Walker M., Selker R., Vick N., Black K., Sisti M., Brem S., Mohr G. (1995). Placebo-controlled trial of safety and efficacy of intraoperative controlled delivery by biodegradable polymers of chemotherapy for recurrent gliomas. Lancet.

[40] Xiao W., Gao H. (2018). The impact of protein corona on the behavior and targeting capability of nanoparticle-based delivery system. Int. J. Pharm..

[41] Hadjidemetriou M., Kostarelos K. (2017). Evolution of the nanoparticle corona. Nat. Nanotechnol..

[42] Caracciolo G., Farokhzad O.C., Mahmoudi M. (2017). Biological Identity of Nanoparticles In Vivo: Clinical Implications of the Protein Corona. Trends Biotechnol..

[43] Bulbake U., Doppalapudi S., Kommineni N., Khan W. (2017). Liposomal Formulations in Clinical Use: An Updated Review. Pharmaceutics.

[44] Gonçalves M., Mignani S., Rodrigues J., Tomás H. (2020). A glance over doxorubicin based-nanotherapeutics: From proof-of-concept studies to solutions in the market. J. Control. Release.

[45] Gabizon A., Shmeeda H., Barenholz Y. (2003). Pharmacokinetics of Pegylated Liposomal Doxorubicin. Clin. Pharmacokinet..

[46] Blank N., Laskov I., Kessous R., Kogan L., Lau S., Sebag I.A., Gotlieb W.H., Rudski L. (2017). Absence of cardiotoxicity with prolonged treatment and large accumulating doses of pegylated liposomal doxorubicin. Cancer Chemother. Pharmacol..

[47] Gabizon A., Shmeeda H., Grenader T. (2012). Pharmacological basis of pegylated liposomal doxorubicin: Impact on cancer therapy. Eur. J. Pharm. Sci..

[48] Bellott R., Auvrignon A., Leblanc T., Pérel Y., Gandemer V., Bertrand Y., Méchinaud F., Bellenger P., Vernois J., Leverger G. (2001). Pharmacokinetics of liposomal daunorubicin (DaunoXome) during a phase I-II study in children with relapsed acute lymphoblastic leukaemia. Cancer Chemother. Pharmacol..

[49] Yeh M.-K., Chang H.-I., Cheng M.-Y. (2011). Clinical development of liposome based drugs: Formulation, characterization, and therapeutic efficacy. Int. J. Nanomed..

[50] Wolfram J., Ferrari M. (2019). Clinical cancer nanomedicine. Nano Today.

[51] Mantripragada S. (2002). A lipid based depot (DepoFoam^®^ technology) for sustained release drug delivery. Prog. Lipid Res..

[52] Gallio E., Richetta E., Finessi M., Stasi M., Pellerito R.E., Bisi G., Ropolo R. (2016). Calculation of tumour and normal tissue biological effective dose in 90 Y liver radioembolization with different dosimetric methods. Phys. Med..

[53] Bastiaannet R., van Roekel C., Smits M.L.J., Elias S.G., van Amsterdam W.A.C., Doan D., Prince J.F., Bruijnen R.C.G., de Jong H.W.A.M., Lam M.G.E.H. (2020). First Evidence for a Dose-Response Relationship in Patients Treated with 166Ho Radioembolization: A Prospective Study. J. Nucl. Med..

[54] Lee E.W., Alanis L., Cho S.-K., Saab S. (2016). Yttrium-90 Selective Internal Radiation Therapy with Glass Microspheres for Hepatocellular Carcinoma: Current and Updated Literature Review. Korean J. Radiol..

[55] Mantry P., Thompson M., Khanna P., Acharya P., Shahin I. (2019). Prolonged Survival With Radioembolization Using Theraspheres in Unresectable HCC. Am. J. Gastroenterol..

[56] Memon K., Lewandowski R.J., Kulik L., Riaz A., Mulcahy M.F., Salem R. (2011). Radioembolization for Primary and Metastatic Liver Cancer. Semin. Radiat. Oncol..

[57] Desai N. (2016). Nanoparticle Albumin-Bound Paclitaxel (Abraxane^®^). Albumin in Medicine.

[58] Bernabeu E., Cagel M., Lagomarsino E., Moretton M., Chiappetta D.A. (2017). Paclitaxel: What has been done and the challenges remain ahead. Int. J. Pharm..

[59] Revel-Mouroz P., Otal P., Jaffro M., Petermann A., Meyrignac O., Rabinel P., Mokrane F.-Z. (2017). Other non-surgical treatments for liver cancer. Rep. Pract. Oncol. Radiother..

[60] Chew S.A., Moscato S., George S., Azimi B., Danti S. (2019). Liver Cancer: Current and Future Trends Using Biomaterials. Cancers.

[61] Liu Y.-S., Ou M.-C., Tsai Y.-S., Lin X.-Z., Wang C.-K., Tsai H.-M., Chuang M.-T. (2015). Transarterial Chemoembolization Using Gelatin Sponges or Microspheres Plus Lipiodol-Doxorubicin versus Doxorubicin-Loaded Beads for the Treatment of Hepatocellular Carcinoma. Korean J. Radiol..

[62] Wright J.C., Tao Leonard S., Stevenson C.L., Beck J.C., Chen G., Jao R.M., Johnson P.A., Leonard J., Skowronski R.J. (2001). An in vivo/in vitro comparison with a leuprolide osmotic implant for the treatment of prostate cancer. J. Control. Release.

[63] Li J., Mooney D.J. (2016). Designing hydrogels for controlled drug delivery. Nat. Rev. Mater..

[64] Shore N. (2010). Introducing Vantas: The First Once-Yearly Luteinising Hormone-Releasing Hormone Agonist. Eur. Urol. Suppl..

[65] Wex J., Sidhu M., Odeyemi I., Abou-Setta A.M., Retsa P., Tombal B. (2013). Leuprolide acetate 1-, 3- and 6-monthly depot formulations in androgen deprivation therapy for prostate cancer in nine European countries: Evidence review and economic evaluation. Clin. Outcomes Res..

[66] Saltzstein D., Shore N.D., Moul J.W., Chu F., Concepcion R., de la Motte S., McLane J.A., Atkinson S., Yang A., Crawford E.D. (2018). Pharmacokinetic and pharmacodynamic comparison of subcutaneous versus intramuscular leuprolide acetate formulations in male subjects. Ther. Adv. Urol..

[67] Chou A.J., Kleinerman E.S., Krailo M.D., Chen Z., Betcher D.L., Healey J.H., Conrad E.U., Nieder M.L., Weiner M.A., Wells R.J. (2009). Addition of muramyl tripeptide to chemotherapy for patients with newly diagnosed metastatic osteosarcoma. Cancer.

[68] Bun S., Yunokawa M., Tamaki Y., Shimomura A., Shimoi T., Kodaira M., Shimizu C., Yonemori K., Fujiwara Y., Makino Y. (2018). Symptom management: The utility of regional cooling for hand-foot syndrome induced by pegylated liposomal doxorubicin in ovarian cancer. Support. Care Cancer.

[69] Kanwal U., Irfan Bukhari N., Ovais M., Abass N., Hussain K., Raza A. (2018). Advances in nano-delivery systems for doxorubicin: An updated insight. J. Drug Target..

[70] Luo R., Li Y., He M., Zhang H., Yuan H., Johnson M., Palmisano M., Zhou S., Sun D. (2017). Distinct biodistribution of doxorubicin and the altered dispositions mediated by different liposomal formulations. Int. J. Pharm..

[71] Batist G., Harris L., Azarnia N., Lee L.W., Daza-Ramirez P. (2006). Improved anti-tumor response rate with decreased cardiotoxicity of non-pegylated liposomal doxorubicin compared with conventional doxorubicin in first-line treatment of metastatic breast cancer in patients who had received prior adjuvant doxorubicin: Resu. Anticancer. Drugs.

[72] Yardley D.A. (2013). nab-Paclitaxel mechanisms of action and delivery. J. Control. Release.

[73] Gradishar W.J. (2006). Albumin-bound paclitaxel: A next-generation taxane. Expert Opin. Pharmacother..

[74] Gardner E.R., Dahut W.L., Scripture C.D., Jones J., Aragon-Ching J.B., Desai N., Hawkins M.J., Sparreboom A., Figg W.D. (2008). Randomized Crossover Pharmacokinetic Study of Solvent-Based Paclitaxel and nab-Paclitaxel. Clin. Cancer Res..

[75] Barkat M.A., Beg S., Pottoo F.H., Ahmad F.J. (2019). Nanopaclitaxel therapy: An evidence based review on the battle for next-generation formulation challenges. Nanomedicine.

[76] Nouri Y.M., Kim J.H., Yoon H.-K., Ko H.-K., Shin J.H., Gwon D. (2019). Il Update on Transarterial Chemoembolization with Drug-Eluting Microspheres for Hepatocellular Carcinoma. Korean J. Radiol..

[77] Chen Y.-P., Zhang J.-L., Zou Y., Wu Y.-L. (2019). Recent Advances on Polymeric Beads or Hydrogels as Embolization Agents for Improved Transcatheter Arterial Chemoembolization (TACE). Front. Chem..

[78] Martin R., Irurzun J., Munchart J., Trofimov I., Scupchenko A., Tatum C., Narayanan G. (2011). Optimal technique and response of doxorubicin beads in hepatocellular cancer: Bead size and dose. Korean J. Hepatol..

[79] Malagari K., Emmanouil E., Pomoni M., Kelekis D. (2014). Chemoembolization with DC Bead^TM^ for the treatment of hepatocellular carcinoma: An update. Hepatic Oncol..

[80] Nicolini A., Martinetti L., Crespi S., Maggioni M., Sangiovanni A. (2010). Transarterial Chemoembolization with Epirubicin-eluting Beads versus Transarterial Embolization before Liver Transplantation for Hepatocellular Carcinoma. J. Vasc. Interv. Radiol..

[81] Song M.J., Park C.-H., Kim J.D., Kim H.Y., Bae S.H., Choi J.Y., Yoon S.K., Chun H.J., Choi B.G., Lee H.G. (2011). Drug-eluting bead loaded with doxorubicin versus conventional Lipiodol-based transarterial chemoembolization in the treatment of hepatocellular carcinoma. Eur. J. Gastroenterol. Hepatol..

[82] Poon R.T.P., Tso W.K., Pang R.W.C., Ng K.K.C., Woo R., Tai K.S., Fan S.T. (2007). A Phase I/II Trial of Chemoembolization for Hepatocellular Carcinoma Using a Novel Intra-Arterial Drug-Eluting Bead. Clin. Gastroenterol. Hepatol..

[83] Malagari K., Pomoni M., Moschouris H., Kelekis A., Charokopakis A., Bouma E., Spyridopoulos T., Chatziioannou A., Sotirchos V., Karampelas T. (2014). Chemoembolization of Hepatocellular Carcinoma with Hepasphere 30–60 μm. Safety and Efficacy Study. Cardiovasc. Intervent. Radiol..

[84] Bouvry C., Palard X., Edeline J., Ardisson V., Loyer P., Garin E., Lepareur N. (2018). Transarterial Radioembolization (TARE) Agents beyond 90 Y-Microspheres. Biomed. Res. Int..

[85] Anselmo A.C., Mitragotri S. (2019). Nanoparticles in the clinic: An update. Bioeng. Transl. Med..

[86] Li R.-J., Jin R., Liu C., Cao X., Manning M.L., Di X.M., Przepiorka D., Namuswe F., Deisseroth A., Goldberg K.B. (2020). FDA Approval Summary: Calaspargase Pegol-mknl For Treatment of Acute Lymphoblastic Leukemia in Children and Young Adults. Clin. Cancer Res..

[87] Lew G. (2020). Space for Calaspargase? A New Asparaginase for Acute Lymphoblastic Leukemia. Clin. Cancer Res..

[88] Vrooman L.M., Blonquist T.M., Supko J.G., Hunt S.K., O’Brien J.E., Kay-Green S., Athale U.H., Clavell L.A., Cole P.D., Harris M.H. (2019). Efficacy and toxicity of pegaspargase and calaspargase pegol in childhood acute lymphoblastic leukemia/lymphoma: Results of DFCI 11-001. J. Clin. Oncol..

[89] Douer D. (2016). Efficacy and Safety of Vincristine Sulfate Liposome Injection in the Treatment of Adult Acute Lymphocytic Leukemia. Oncologist.

[90] Moore A., Soosay Raj T., Smith A. (2013). Vincristine sulfate liposomal injection for acute lymphoblastic leukemia. Int. J. Nanomed..

[91] Silverman J.A., Deitcher S.R. (2013). Marqibo^®^ (vincristine sulfate liposome injection) improves the pharmacokinetics and pharmacodynamics of vincristine. Cancer Chemother. Pharmacol..

[92] Passero F.C., Grapsa D., Syrigos K.N., Saif M.W. (2016). The safety and efficacy of Onivyde (irinotecan liposome injection) for the treatment of metastatic pancreatic cancer following gemcitabine-based therapy. Expert Rev. Anticancer. Ther..

[93] Zhang H. (2016). Onivyde for the therapy of multiple solid tumors. Onco Targets Ther..

[94] Alfayez M., Kantarjian H., Kadia T., Ravandi-Kashani F., Daver N. (2020). CPX-351 (vyxeos) in AML. Leuk. Lymphoma.

[95] Blair H.A. (2018). Daunorubicin/Cytarabine Liposome: A Review in Acute Myeloid Leukaemia. Drugs.

[96] Chen E.C., Fathi A.T., Brunner A.M. (2018). Reformulating acute myeloid leukemia: Liposomal cytarabine and daunorubicin (CPX-351) as an emerging therapy for secondary AML. OncoTargets Ther..

[97] Chen K.T.J., Gilabert-Oriol R., Bally M.B., Leung A.W.Y. (2019). Recent Treatment Advances and the Role of Nanotechnology, Combination Products, and Immunotherapy in Changing the Therapeutic Landscape of Acute Myeloid Leukemia. Pharm. Res..

[98] Germain M., Caputo F., Metcalfe S., Tosi G., Spring K., Åslund A.K.O., Pottier A., Schiffelers R., Ceccaldi A., Schmid R. (2020). Delivering the power of nanomedicine to patients today. J. Control. Release.

[99] Lancet J.E., Uy G.L., Cortes J.E., Newell L.F., Lin T.L., Ritchie E.K., Stuart R.K., Strickland S.A., Hogge D., Solomon S.R. (2018). CPX-351 (cytarabine and daunorubicin) Liposome for Injection Versus Conventional Cytarabine Plus Daunorubicin in Older Patients With Newly Diagnosed Secondary Acute Myeloid Leukemia. J. Clin. Oncol..

[100] Tzogani K., Penttilä K., Lapveteläinen T., Hemmings R., Koenig J., Freire J., Márcia S., Cole S., Coppola P., Flores B. (2020). EMA Review of Daunorubicin and Cytarabine Encapsulated in Liposomes (Vyxeos, CPX-351) for the Treatment of Adults with Newly Diagnosed, Therapy-Related Acute Myeloid Leukemia or Acute Myeloid Leukemia with Myelodysplasia-Related Changes. Oncologist.

[101] Borgå O., Lilienberg E., Bjermo H., Hansson F., Heldring N., Dediu R. (2019). Pharmacokinetics of Total and Unbound Paclitaxel After Administration of Paclitaxel Micellar or Nab-Paclitaxel: An Open, Randomized, Cross-Over, Explorative Study in Breast Cancer Patients. Adv. Ther..

[102] Guiu B., Schmitt A., Reinhardt S., Fohlen A., Pohl T., Wendremaire M., Denys A., Blümmel J., Boulin M. (2015). Idarubicin-Loaded ONCOZENE Drug-Eluting Embolic Agents for Chemoembolization of Hepatocellular Carcinoma: In Vitro Loading and Release and In Vivo Pharmacokinetics. J. Vasc. Interv. Radiol..

[103] Delicque J., Guiu B., Boulin M., Schwanz H., Piron L., Cassinotto C. (2018). Liver chemoembolization of hepatocellular carcinoma using TANDEM^®^ microspheres. Futur. Oncol..

[104] Richter G., Radeleff B., Stroszczynski C., Pereira P., Helmberger T., Barakat M., Huppert P. (2018). Safety and Feasibility of Chemoembolization with Doxorubicin-Loaded Small Calibrated Microspheres in Patients with Hepatocellular Carcinoma: Results of the MIRACLE I Prospective Multicenter Study. Cardiovasc. Interv. Radiol..

[105] Aliberti C., Carandina R., Sarti D., Mulazzani L., Pizzirani E., Guadagni S., Fiorentini G. (2017). Chemoembolization Adopting Polyethylene Glycol Drug-Eluting Embolics Loaded With Doxorubicin for the Treatment of Hepatocellular Carcinoma. Am. J. Roentgenol..

[106] Aliberti C., Carandina R., Sarti D., Mulazzani L., Catalano V., Felicioli A., Coschiera P., Fiorentini G. (2016). Hepatic arterial infusion of polyethylene glycol drug-eluting beads for primary and metastatic liver cancer therapy. Anticancer. Res..

[107] Pottier A., Borghi E., Levy L. (2015). Metals as radio-enhancers in oncology: The industry perspective. Biochem. Biophys. Res. Commun..

[108] Reinders M.T.M., Smits M.L.J., van Roekel C., Braat A.J.A.T. (2019). Holmium-166 Microsphere Radioembolization of Hepatic Malignancies. Semin. Nucl. Med..

[109] Bonvalot S., Rutkowski P.L., Thariat J., Carrère S., Ducassou A., Sunyach M.-P., Agoston P., Hong A., Mervoyer A., Rastrelli M. (2019). NBTXR3, a first-in-class radioenhancer hafnium oxide nanoparticle, plus radiotherapy versus radiotherapy alone in patients with locally advanced soft-tissue sarcoma (Act.In.Sarc): A multicentre, phase 2–3, randomised, controlled trial. Lancet Oncol..

[110] Bisso S., Leroux J.-C. (2020). Nanopharmaceuticals: A focus on their clinical translatability. Int. J. Pharm..

[111] Chajon E., Pracht M., De Baere T., Nguyen F., Bronowicki J.-P., Vendrely V., Baumann A.-S., Croisé-Laurent V., Deutsch E. (2018). A phase I/II trial of NBTXR3 nanoparticles activated by SBRT in the treatment of liver cancers. J. Clin. Oncol..

[112] Bonvalot S., Le Pechoux C., De Baere T., Buy X., Italiano A., Stockle E., Terrier P., Lassau N., Le Cesne A., Sargos P. (2014). Phase I study of NBTXR3 nanoparticles, in patients with advanced soft tissue sarcoma (STS). J. Clin. Oncol..

[113] Bonvalot S., Le Pechoux C., De Baere T., Kantor G., Buy X., Stoeckle E., Terrier P., Sargos P., Coindre J.M., Lassau N. (2017). First-in-Human Study Testing a New Radioenhancer Using Nanoparticles (NBTXR3) Activated by Radiation Therapy in Patients with Locally Advanced Soft Tissue Sarcomas. Clin. Cancer Res..

[114] Grauer O., Jaber M., Hess K., Weckesser M., Schwindt W., Maring S., Wölfer J., Stummer W. (2019). Combined intracavitary thermotherapy with iron oxide nanoparticles and radiotherapy as local treatment modality in recurrent glioblastoma patients. J. Neurooncol..

[115] Mahmoudi K., Bouras A., Bozec D., Ivkov R., Hadjipanayis C. (2018). Magnetic hyperthermia therapy for the treatment of glioblastoma: A review of the therapy’s history, efficacy and application in humans. Int. J. Hyperth..

[116] Martinelli C., Pucci C., Ciofani G. (2019). Nanostructured carriers as innovative tools for cancer diagnosis and therapy. APL Bioeng..

[117] El-Boubbou K. (2018). Magnetic iron oxide nanoparticles as drug carriers: Clinical relevance. Nanomedicine.

[118] Abou-Alfa G.K., Qin S., Ryoo B.-Y., Lu S.-N., Yen C.-J., Feng Y.-H., Lim H.Y., Izzo F., Colombo M., Sarker D. (2018). Phase III randomized study of second line ADI-PEG 20 plus best supportive care versus placebo plus best supportive care in patients with advanced hepatocellular carcinoma. Ann. Oncol..

[119] Doherty G.J., Tempero M., Corrie P.G. (2018). HALO-109–301: A Phase III trial of PEGPH20 (with gemcitabine and nab-paclitaxel) in hyaluronic acid-high stage IV pancreatic cancer. Futur. Oncol..

[120] Naing A., Papadopoulos K.P., Autio K.A., Ott P.A., Patel M.R., Wong D.J., Falchook G.S., Pant S., Whiteside M., Rasco D.R. (2016). Safety, Antitumor Activity, and Immune Activation of Pegylated Recombinant Human Interleukin-10 (AM0010) in Patients With Advanced Solid Tumors. J. Clin. Oncol..

[121] Butts C., Socinski M.A., Mitchell P.L., Thatcher N., Havel L., Krzakowski M., Nawrocki S., Ciuleanu T.-E., Bosquée L., Trigo J.M. (2014). Tecemotide (L-BLP25) versus placebo after chemoradiotherapy for stage III non-small-cell lung cancer (START): A randomised, double-blind, phase 3 trial. Lancet Oncol..

[122] Zisman N., Dos Santos N., Johnstone S., Tsang A., Bermudes D., Mayer L., Tardi P. (2011). Optimizing Liposomal Cisplatin Efficacy through Membrane Composition Manipulations. Chemother. Res. Pract..

[123] Chauffert B., Mornex F., Bonnetain F., Rougier P., Mariette C., Bouché O., Bosset J.F., Aparicio T., Mineur L., Azzedine A. (2008). Phase III trial comparing intensive induction chemoradiotherapy (60 Gy, infusional 5-FU and intermittent cisplatin) followed by maintenance gemcitabine with gemcitabine alone for locally advanced unresectable pancreatic cancer. Definitive results of the 2. Ann. Oncol..

[124] Osada A. (2019). NC-6004, a novel cisplatin nanoparticle, in combination with pembrolizumab for head and neck cancer. Integr. Clin. Med..

[125] Dunne M., Epp-Ducharme B., Sofias A.M., Regenold M., Dubins D.N., Allen C. (2019). Heat-activated drug delivery increases tumor accumulation of synergistic chemotherapies. J. Control. Release.

[126] Wood B.J., Poon R.T., Locklin J.K., Dreher M.R., Ng K.K., Eugeni M., Seidel G., Dromi S., Neeman Z., Kolf M. (2012). Phase I Study of Heat-Deployed Liposomal Doxorubicin during Radiofrequency Ablation for Hepatic Malignancies. J. Vasc. Interv. Radiol..

[127] Merle P., Blanc J.-F., Phelip J.-M., Pelletier G., Bronowicki J.-P., Touchefeu Y., Pageaux G., Gerolami R., Habersetzer F., Nguyen-Khac E. (2019). Doxorubicin-loaded nanoparticles for patients with advanced hepatocellular carcinoma after sorafenib treatment failure (RELIVE): A phase 3 randomised controlled trial. Lancet Gastroenterol. Hepatol..

[128] Ignatiadis M., Zardavas D., Lemort M., Wilke C., Vanderbeeken M.-C., D’Hondt V., De Azambuja E., Gombos A., Lebrun F., Dal Lago L. (2016). Feasibility Study of EndoTAG-1, a Tumor Endothelial Targeting Agent, in Combination with Paclitaxel followed by FEC as Induction Therapy in HER2-Negative Breast Cancer. PLoS ONE.

[129] Negishi T., Koizumi F., Uchino H., Kuroda J., Kawaguchi T., Naito S., Matsumura Y. (2006). NK105, a paclitaxel-incorporating micellar nanoparticle, is a more potent radiosensitising agent compared to free paclitaxel. Br. J. Cancer.

[130] Fujiwara Y., Mukai H., Saeki T., Ro J., Lin Y.-C., Nagai S.E., Lee K.S., Watanabe J., Ohtani S., Kim S.B. (2019). A multi-national, randomised, open-label, parallel, phase III non-inferiority study comparing NK105 and paclitaxel in metastatic or recurrent breast cancer patients. Br. J. Cancer.

[131] Perez E.A., Awada A., O’Shaughnessy J., Rugo H.S., Twelves C., Im S.-A., Gómez-Pardo P., Schwartzberg L.S., Diéras V., Yardley D.A. (2015). Etirinotecan pegol (NKTR-102) versus treatment of physician’s choice in women with advanced breast cancer previously treated with an anthracycline, a taxane, and capecitabine (BEACON): A randomised, open-label, multicentre, phase 3 trial. Lancet Oncol..

[132] Tripathy D., Tolaney S.M., Seidman A.D., Anders C.K., Ibrahim N., Rugo H.S., Twelves C., Dieras V., Müller V., Tagliaferri M. (2019). ATTAIN: Phase III study of etirinotecan pegol versus treatment of physician’s choice in patients with metastatic breast cancer and brain metastases. Futur. Oncol..

[133] Shen X., Li T., Xie X., Feng Y., Chen Z., Yang H., Wu C., Deng S., Liu Y. (2020). PLGA-Based Drug Delivery Systems for Remotely Triggered Cancer Therapeutic and Diagnostic Applications. Front. Bioeng. Biotechnol..

[134] Li C., Wang J., Wang Y., Gao H., Wei G., Huang Y., Yu H., Gan Y., Wang Y., Mei L. (2019). Recent progress in drug delivery. Acta Pharm. Sin. B.

[135] Pillai G. (2014). Nanomedicines for Cancer Therapy: An Update of FDA Approved and Those under Various Stages of Development. SOJ Pharm. Pharm. Sci..

[136] Baeza A. (2020). Tumor Targeted Nanocarriers for Immunotherapy. Molecules.

[137] Pearce A.K., O’Reilly R.K. (2019). Insights into Active Targeting of Nanoparticles in Drug Delivery: Advances in Clinical Studies and Design Considerations for Cancer Nanomedicine. Bioconjug. Chem..

[138] You J., Li X., de Cui F., Du Y.-Z., Yuan H., Hu F.Q. (2008). Folate-conjugated polymer micelles for active targeting to cancer cells: Preparation, in vitro evaluation of targeting ability and cytotoxicity. Nanotechnology.

[139] Landeros-Martínez L.-L., Glossman-Mitnik D., Flores-Holguín N. (2018). Studying the chemical reactivity properties of the target tumor-environment tripeptides NGR (asparagine-glycine-arginine) and RGD (arginine-glycine-aspartic acid) in their interactions with tamoxifen through conceptual density functional theory. J. Mol. Model..

[140] Hong M., Zhu S., Jiang Y., Tang G., Pei Y. (2009). Efficient tumor targeting of hydroxycamptothecin loaded PEGylated niosomes modified with transferrin. J. Control. Release.

[141] Zhai J., Scoble J.A., Li N., Lovrecz G., Waddington L.J., Tran N., Muir B.W., Coia G., Kirby N., Drummond C.J. (2015). Epidermal growth factor receptor-targeted lipid nanoparticles retain self-assembled nanostructures and provide high specificity. Nanoscale.

[142] Smith J.E., Medley C.D., Tang Z., Shangguan D., Lofton C., Tan W. (2007). Aptamer-Conjugated Nanoparticles for the Collection and Detection of Multiple Cancer Cells. Anal. Chem..

[143] Arranja A.G., Pathak V., Lammers T., Shi Y. (2017). Tumor-targeted nanomedicines for cancer theranostics. Pharmacol. Res..

[144] Mi P. (2020). Stimuli-responsive nanocarriers for drug delivery, tumor imaging, therapy and theranostics. Theranostics.

[145] Cao Z., Li W., Liu R., Li X., Li H., Liu L., Chen Y., Lv C., Liu Y. (2019). pH- and enzyme-triggered drug release as an important process in the design of anti-tumor drug delivery systems. Biomed. Pharmacother..

[146] Liu J., Huang Y., Kumar A., Tan A., Jin S., Mozhi A., Liang X.-J. (2014). pH-Sensitive nano-systems for drug delivery in cancer therapy. Biotechnol. Adv..

[147] Gao G.H., Park M.J., Li Y., Im G.H., Kim J.-H., Kim H.N., Lee J.W., Jeon P., Bang O.Y., Lee J.H. (2012). The use of pH-sensitive positively charged polymeric micelles for protein delivery. Biomaterials.

[148] Li Z.-Y., Hu J.-J., Xu Q., Chen S., Jia H.-Z., Sun Y.-X., Zhuo R.-X., Zhang X.-Z. (2015). A redox-responsive drug delivery system based on RGD containing peptide-capped mesoporous silica nanoparticles. J. Mater. Chem. B.

[149] Xin X., Teng C., Du X., Lv Y., Xiao Q., Wu Y., He W., Yin L. (2018). Drug-delivering-drug platform-mediated potent protein therapeutics via a non-endo-lysosomal route. Theranostics.

[150] Fu H., Shi K., Hu G., Yang Y., Kuang Q., Lu L., Zhang L., Chen W., Dong M., Chen Y. (2015). Tumor-Targeted Paclitaxel Delivery and Enhanced Penetration Using TAT-Decorated Liposomes Comprising Redox-Responsive Poly(Ethylene Glycol). J. Pharm. Sci..

[151] Alsehli M. (2020). Polymeric nanocarriers as stimuli-responsive systems for targeted tumor (cancer) therapy: Recent advances in drug delivery. Saudi Pharm. J..

[152] Chiang C.-S., Shen Y.-S., Liu J.-J., Shyu W.-C., Chen S.-Y. (2016). Synergistic Combination of Multistage Magnetic Guidance and Optimized Ligand Density in Targeting a Nanoplatform for Enhanced Cancer Therapy. Adv. Healthc. Mater..

[153] Farzin A., Etesami S.A., Quint J., Memic A., Tamayol A. (2020). Magnetic Nanoparticles in Cancer Therapy and Diagnosis. Adv. Healthc. Mater..

[154] Mura S., Nicolas J., Couvreur P. (2013). Stimuli-responsive nanocarriers for drug delivery. Nat. Mater..

[155] Al-Ahmady Z.S., Al-Jamal W.T., Bossche J.V., Bui T.T., Drake A.F., Mason A.J., Kostarelos K. (2012). Lipid–Peptide Vesicle Nanoscale Hybrids for Triggered Drug Release by Mild Hyperthermia in Vitro and in Vivo. ACS Nano.

[156] Karimi M., Ghasemi A., Sahandi Zangabad P., Rahighi R., Moosavi Basri S.M., Mirshekari H., Amiri M., Shafaei Pishabad Z., Aslani A., Bozorgomid M. (2016). Smart micro/nanoparticles in stimulus-responsive drug/gene delivery systems. Chem. Soc. Rev..

[157] Luo D., Carter K.A., Razi A., Geng J., Shao S., Giraldo D., Sunar U., Ortega J., Lovell J.F. (2016). Doxorubicin encapsulated in stealth liposomes conferred with light-triggered drug release. Biomaterials.

[158] Aryal M., Arvanitis C.D., Alexander P.M., McDannold N. (2014). Ultrasound-mediated blood–brain barrier disruption for targeted drug delivery in the central nervous system. Adv. Drug Deliv. Rev..

[159] Nam J., Son S., Park K.S., Zou W., Shea L.D., Moon J.J. (2019). Cancer nanomedicine for combination cancer immunotherapy. Nat. Rev. Mater..

[160] Wang J., Zhang Y., Jin N., Mao C., Yang M. (2019). Protein-Induced Gold Nanoparticle Assembly for Improving the Photothermal Effect in Cancer Therapy. ACS Appl. Mater. Interfaces.

[161] Chen Q., Wang C., Cheng L., He W., Cheng Z., Liu Z. (2014). Protein modified upconversion nanoparticles for imaging-guided combined photothermal and photodynamic therapy. Biomaterials.

[162] Aparicio-Blanco J., Torres-Suárez A.I. (2018). Towards tailored management of malignant brain tumors with nanotheranostics. Acta Biomater..

[163] Sonali, Viswanadh M.K., Singh R.P., Agrawal P., Mehata A.K., Pawde D.M., Narendra, Sonkar R., Muthu M.S. (2018). Nanotheranostics: Emerging Strategies for Early Diagnosis and Therapy of Brain Cancer. Nanotheranostics.

[164] Li C., Cao L., Zhang Y., Yi P., Wang M., Tan B., Deng Z., Wu D., Wang Q. (2015). Preoperative Detection and Intraoperative Visualization of Brain Tumors for More Precise Surgery: A New Dual-Modality MRI and NIR Nanoprobe. Small.

[165] Lungu I.I., Grumezescu A.M., Volceanov A., Andronescu E. (2019). Nanobiomaterials Used in Cancer Therapy: An Up-To-Date Overview. Molecules.

[166] Zhou M., Wei W., Chen X., Xu X., Zhang X., Zhang X. (2019). pH and redox dual responsive carrier-free anticancer drug nanoparticles for targeted delivery and synergistic therapy. Nanomed. Nanotechnol. Biol. Med..

[167] Zhu R., He H., Liu Y., Cao D., Yan J., Duan S., Chen Y., Yin L. (2019). Cancer-Selective Bioreductive Chemotherapy Mediated by Dual Hypoxia-Responsive Nanomedicine upon Photodynamic Therapy-Induced Hypoxia Aggravation. Biomacromolecules.

[168] Fowler J.E. (2001). Patient-reported experience with the Viadur 12-month leuprolide implant for prostate cancer. Urology.

[169] Kanwar N., Sinha V.R. (2019). In situ forming depot as sustained-release drug delivery systems. Crit. Rev. Ther. Drug Carr. Syst..

[170] Salvioni L., Rizzuto M.A., Bertolini J.A., Pandolfi L., Colombo M., Prosperi D. (2019). Thirty years of cancer nanomedicine: Success, frustration, and hope. Cancers.

[171] Rafiyath S.M., Rasul M., Lee B., Wei G., Lamba G., Liu D. (2012). Comparison of safety and toxicity of liposomal doxorubicin vs. conventional anthracyclines: A meta-analysis. Exp. Hematol. Oncol..

[172] Hua S., de Matos M.B.C., Metselaar J.M., Storm G. (2018). Current trends and challenges in the clinical translation of nanoparticulate nanomedicines: Pathways for translational development and commercialization. Front. Pharmacol..

[173] Hussain Z., Khan S., Imran M., Sohail M., Shah S.W.A., de Matas M. (2019). PEGylation: A promising strategy to overcome challenges to cancer-targeted nanomedicines: A review of challenges to clinical transition and promising resolution. Drug Deliv. Transl. Res..

[174] Fang Y., Xue J., Gao S., Lu A., Yang D., Jiang H., He Y., Shi K. (2017). Cleavable PEGylation: A strategy for overcoming the “PEG dilemma” in efficient drug delivery. Drug Deliv..

[175] He H., Liu L., Morin E.E., Liu M., Schwendeman A. (2019). Survey of Clinical Translation of Cancer Nanomedicines—Lessons Learned from Successes and Failures. Acc. Chem. Res..

[176] Yang J.D., Hainaut P., Gores G.J., Amadou A., Plymoth A., Roberts L.R. (2019). A global view of hepatocellular carcinoma: Trends, risk, prevention and management. Nat. Rev. Gastroenterol. Hepatol..

[177] Drug-Eluting Beads|Radiology Key. https://radiologykey.com/drug-eluting-beads/.

